# VdPex30, a peroxisomal membrane protein, is involved in carbon metabolism, stress response, and pathogenicity of *Verticillium dahliae*

**DOI:** 10.1128/spectrum.00017-26

**Published:** 2026-05-18

**Authors:** Zhiheng Zhang, Xiaoqing Liu, Wenfang Guo, Huiming Guo, Hongmei Cheng, Xiaoxiao Zhang, Xiaofeng Su

**Affiliations:** 1 Guangxi Key Laboratory of Agro-environment and Agro-product Safety, College of Agriculture, Guangxi University 12664, Nanning, China; 2 National Key Laboratory of Agricultural Microbiology, Biotechnology Research Institute, Chinese Academy of Agricultural Sciences 471740 , Beijing, China; 3 Key Laboratory of Agricultural Microbiome (MARA), Chinese Academy of Agricultural Sciences 12661, Beijing, China; 4 National Nanfan Research Institute, Chinese Academy of Agricultural Sciences, Sanya, China; National Taiwan University, Taipei, Taiwan

**Keywords:** *Verticillium dahliae*, peroxisome, fungal pathogenicity, host-induced gene silencing, stress response

## Abstract

The fungus *Verticillium dahliae* causes Verticillium wilt in plants and has a broad host range. The peroxisomal membrane protein gene *VdPex30* (VDAG_09094), identified in a host-induced gene silencing screen, plays a crucial role in fungal pathogenicity. *VdPex30* encodes a 671-amino-acid protein of the Pex24p subfamily, with expression strongly induced during early infection. *VdPex30***-**RNA interference transgenic tobacco showed enhanced resistance to *V. dahliae*. Δ*VdPex30* mutant strains displayed impaired growth, sporulation, and tolerance to oxidative, cell wall, and osmotic stresses, along with reduced virulence on cotton, and all these defects were restored by complementation. The *ΔVdPex30* strains were hypersensitive to H_₂_O_₂_, accumulated higher reactive oxygen species, and downregulated oxidative response-related genes. Transcriptome analysis identified 3,623 differentially expressed genes (DEGs), confirmed by reverse transcription quantitative PCR (RT-qPCR), and a subset of representative DEGs was further validated by RT-qPCR. Moreover, VdMIase (VDAG_01341) was identified as a novel VdPex30-interacting protein. These findings reveal that VdPex30 is vital for growth, stress adaptation, and pathogenicity of *V. dahliae*, providing a potential target for Verticillium wilt control.

**IMPORTANCE:**

*Verticillium dahliae* causes Verticillium wilt, a destructive vascular disease that severely threatens many crops, including cotton. Identifying fungal genes required for infection can enable more durable control strategies. Here, we demonstrate that VdPex30, a peroxisomal membrane protein identified in a host-induced gene silencing screen, is essential for fungal growth, stress adaptation, and pathogenicity. *VdPex30* knockout (Δ*VdPex30*) strains showed impaired carbon utilization and sporulation, hypersensitivity to oxidative stress, elevated intracellular reactive oxygen species, and markedly reduced virulence on cotton, all of which were restored by genetic complementation. Moreover, plant-mediated RNA interference targeting the fungal *VdPex30* transcript enhanced host resistance to infection. Together, our findings reveal a critical link between peroxisome function and fungal virulence and highlight *VdPex30* as a promising target for RNA interference-based management of Verticillium wilt.

## INTRODUCTION

*Verticillium dahliae* has an exceptionally broad host range, infecting over 660 plant species across 38 families (1). Global assessments estimate that Verticillium wilt (VW), caused by *V. dahliae*, leads to direct annual economic losses exceeding 100 billion USD worldwide (2, 3). This fungus first persists in soil as microsclerotia, which germinate under favorable conditions, attach to plant roots via chemotaxis, and invade through multiple pathways (4). Upon invasion, it secretes a variety of degrading enzymes to disrupt cellular structures, proliferates extensively within vascular bundles to block conductive channels, and simultaneously produces toxins that interfere with plant physiological metabolism (5). Furthermore, it engages in intricate molecular interplay with host plants to evade immune defenses, with multiple coordinated processes collectively contributing to plant pathogenesis (6–10). Currently, the most effective strategy to control *V. dahliae* is breeding resistant cultivars (11). However, conventional cotton breeding is a time-consuming process, which often limits the timely deployment of resistant cultivars in the field (4). Therefore, elucidating the molecular basis of *V. dahliae* pathogenicity is crucial for innovating sustainable disease management approaches.

Host-induced gene silencing (HIGS) is a novel plant disease resistance strategy based on the RNA interference (RNAi) mechanism (12). By expressing double-stranded RNAs (dsRNAs) or small interfering RNAs in the host plant, HIGS mediates sequence-specific degradation of essential pathogen transcripts, effectively suppressing fungal growth, virulence, and colonization (13). HIGS has been successfully employed to inhibit the development of the powdery mildew fungus *Blumeria graminis* in barley and to suppress the infection and colonization of *Puccinia triticina* (wheat leaf rust) and *Fusarium graminearum* (Fusarium head blight) in wheat (14–16). Recent independent studies have demonstrated the effectiveness of the HIGS strategy in silencing key pathogenicity genes of *V. dahliae*, highlighting its potential as a promising tool for functional genomics and VW management in crops (17).

Carbon source utilization constitutes a fundamental requirement for fungal survival, with distinct species exhibiting preferential assimilation of specific substrates that critically modulate growth rates (18). Carbon availability further governs pathogenicity through structural remodeling of the fungal cell wall. In *Candida albicans*, carbon sources directly influence the relative abundance of key structural components: glucose supplementation promotes rigid wall architectures enriched in chitin and β-glucans, whereas alternative carbon sources (e.g., ethanol) yield looser assemblies. These carbon-dependent modifications dictate mechanical integrity, stress tolerance, and host-pathogen interactions (19). Pathogens infecting plant hosts similarly rely on carbon metabolism for virulence; in poplar-infecting *Botryosphaeria* and *Valsa* spp., significant downregulation of carbon metabolic and transporter genes during early infection impairs growth and pathogenic capacity (20). Thus, carbon source acquisition and assimilation represent pivotal determinants of fungal fitness and virulence.

Peroxisomes are ubiquitous single-membrane organelles critical for cellular metabolism and physiological homeostasis in eukaryotic cells (21, 22). They harbor abundant catalase and peroxidases that regulate reactive oxygen species (ROS), thereby maintaining ROS balance and mitigating oxidative stress (23, 24). Through peroxisomal β-oxidation, they catabolize long-chain fatty acids into acetyl-CoA to fuel cellular energy production (25). Additionally, enzymes localized within peroxisomes catalyze the synthesis of plasmalogens by generating alkyl-dihydroxyacetone phosphate, a key intermediate subsequently processed in the endoplasmic reticulum to ensure membrane stability and functional integrity (26). Collectively, these functions establish peroxisomes as indispensable organelles for cellular vitality.

Peroxisomes critically influence fungal pathogenicity through multifaceted mechanisms. In *Magnaporthe oryzae*, peroxisomal β-oxidation degrades fatty acids to fuel germ tube development and energy metabolism (27). Functional integrity of peroxisomal membrane proteins—regulated by peroxins (Pex proteins)—ensures peroxisome biogenesis and activity (28–30). Core components *Pex3* and *Pex19* mediate peroxisomal membrane assembly and protein targeting (31, 32). Disruption of this machinery impairs virulence: *Colletotrichum orbiculare* Δ*CoPex13* mutant strains exhibit defective fatty acid metabolism, melanin biosynthesis, and turgor generation, compromising host penetration (33), while *MoPex7*-deficient *M. oryzae* shows impaired lipid utilization and attenuated virulence (34). Peroxisomes also generate Woronin bodies that seal septal pores to maintain cellular integrity during infection (35–37). Crucially, peroxisomes counteract host-derived oxidative bursts by regulating ROS homeostasis. Mutants lacking key peroxins (e.g., Δ*MoPex14/17* in *M. oryzae* [38] and Δ*AoPEX14/17* in *Arthrobotrys oligospora* [35]) display hypersensitivity to ROS, aberrant development, lipid/ROS accumulation, and reduced virulence. Notably, Δ*MoPex6* abolishes infection structure formation and pathogenicity due to defective peroxisome function (39).

In our prior HIGS screen identifying 92 candidate virulence factors of *V. dahliae*, transient silencing of *VdPex30* (VDAG_09094) significantly compromised pathogenicity, though its functional role remained uncharacterized (17). Herein, we demonstrated that *VdPex30* was essential for the pathogenic factor in *V. dahliae* using HIGS, RNAi-transgenic tobacco, and *VdPex30*-knockout mutant strains (Δ*VdPex30*). Δ*VdPex30* strains exhibited high sensitivity to H_₂_O_₂_ and elevated intracellular ROS levels, accompanied by downregulated expression of oxidative response-related genes. Furthermore, *VdPex30* mediated carbon source utilization and maintained cell wall/membrane integrity under stress. RNA-seq revealed that *VdPex30* deletion broadly dysregulated transcriptional networks governing diverse biological processes. Critically, yeast two-hybrid (Y2H), luciferase complementation (LUC), and bimolecular fluorescence complementation (BiFC) assays confirmed a physical interaction between VdPex30 and methylitaconate delta2-delta3-isomerase (VdMIase, VDAG_01341). The study provides a novel candidate target for RNAi-based disease resistance and deepens our understanding of peroxisome-related pathogenicity mechanisms in *V. dahliae*.

## MATERIALS AND METHODS

### Gene annotation

Gene annotation numbers (e.g., VDAG_09094) were derived from the *V. dahliae* VdLs.17 genome annotation (40).

### Multiple sequence alignment

Protein sequences of VdPex30 and its homologs from representative species were retrieved from public databases. Multiple sequence alignment was performed using the ClustalW program implemented in MEGA (v7.0.26) (41) with default parameters. The aligned sequences were imported into ESPript 3.0 (42) to generate the alignment figure and highlight conserved residues based on the alignment output.

### Transmembrane domain prediction

Transmembrane helices were predicted using TMHMM (v2.0) with default parameters. Regions with a predicted transmembrane probability >0.95 were considered transmembrane segments (43). In addition, regions with a probability >0.85 were regarded as putative atypical transmembrane regions.

### Phylogenetic analysis

Protein sequences of Pex24p family members from different species were retrieved from UniProt. The phylogenetic tree was constructed in MEGA (v7.0.26) using the neighbor-joining method with default parameters. The resulting tree was visualized and annotated in MEGA.

### Plant materials and fungal strain growth conditions

Upland cotton (*Gossypium hirsutum* L.) Coker 312 plants were cultivated in pots containing a sterilized mixture of peat compost and vermiculite at a 1:1 ratio. The greenhouse environment was carefully controlled, maintaining a temperature of 25°C, relative humidity levels between 60% and 75%, and a photoperiod of 16 h of light followed by 8 h of darkness. For the infection assay and observation of disease symptoms, seedlings at the two-true-leaf stage were selected.

A defoliating strain of the pathogenic fungus *V. dahliae* (V991) was acquired from Professor Guiliang Jian of the Institute of Plant Protection at the Chinese Academy of Agricultural Sciences in Beijing, China. This strain was cultured on potato dextrose agar (PDA), comprising 200 g L^−1^ potato, 20 g L^−1^ glucose, and 20 g L^−1^ agar, supplemented with 50 mg L^−1^ ampicillin (Amp) and 50 mg L^−1^ kanamycin (Kan). The cultures were maintained at 25°C for a period of 5–7 days. Additionally, the strain was grown in complete medium (CM), which contained 6 g L^−1^ yeast extract, 6 g L^−1^ casein acid hydrolysate, and 10 g L^−1^ sucrose, with the same concentrations of Amp and Kan. This incubation took place at 25°C for 2–3 days.

### Plasmid construction

To obtain cDNA for subsequent plasmid construction, *V. dahliae* was cultured in CM for a duration of 2–3 days. Subsequently, the mycelium was harvested, frozen in liquid nitrogen, and ground into a fine powder using a high-throughput tissue grinder (Tiangen, Beijing, China). Total RNA extraction was performed utilizing the RNA Easy Fast Tissue Kit (Tiangen, Beijing, China), in accordance with the manufacturer’s established protocols. The concentration of the extracted RNA was quantitatively analyzed using a NanoDrop ND-100 spectrophotometer (Thermo Fisher Scientific, Waltham, MA, USA), while the integrity and quality of the RNA were evaluated through agarose gel electrophoresis. A 1 μg aliquot of total RNA was employed for the synthesis of first-strand complementary DNA (cDNA) using the HiScript III 1st Strand cDNA Synthesis Kit (+gDNA wiper) (Vazyme, Nanjing, China). The resultant cDNA was subsequently stored at −20°C for future applications.

Gateway cloning technology was employed to construct a plasmid for the expression of dsRNA targeting *VdPex30*. The target gene was amplified using the primers *VdPex30*-1-F and *VdPex30*-1-R (Table S1) and subsequently cloned into the intermediate vector pDONR207. An expression cassette, designated as pK7GWIWG2(I)::*VdPex30*, was created via an attL to attR (LR) recombination reaction between the entry plasmid pDONR207::*VdPex30* and the destination vector pK7GWIWG2(I), utilizing the Gateway LR Clonase II Enzyme Mix (Thermo Fisher Scientific, USA). Positive clones were identified and selected for subsequent preparation of the RNAi vector, following previously established protocols. The RNAi plasmids were then transformed into *Agrobacterium tumefaciens* LBA4404 cells for further analysis.

### Pathogenicity assay

After incubation in CM for 2–3 days, spores were collected, counted using a hemocytometer, and diluted with sterile water to a final concentration of approximately 1 × 10⁷ spores mL^−1^. Cotton seedlings at the two-true-leaf stage were removed from the soil, and then the roots were dipped for 5 min before replanting. Disease symptoms were recorded 14 days after inoculation with the V991 strain. Disease severity was assessed at 14 days post-inoculation (dpi) using a 0–4 disease index (DI) scale based on leaf wilting and chlorosis symptoms: 0, no visible symptoms; 1, both cotyledons yellowing or wilting while true leaves remained symptomless; 2, both cotyledons symptomatic and 20%–50% of true leaves showing yellowing or wilting; 3, 50%–75% of leaves (including cotyledons) showing yellowing or wilting; and 4, all leaves symptomatic, leaf abscission, or plant death. The disease index (%) was calculated as follows: DI (%) = [∑(disease grade × number of plants at that grade)/(total number of assessed plants × 4)] × 100. To assess the extent of vascular tissue browning, we used a stereoscope (MVX10; Olympus, Tokyo, Japan) to observe the stems of plants infected with *Vd* strains following their dissection.

### RNA extraction and RT-qPCR analysis

Total RNA was extracted from *V. dahliae*-infected cotton roots at 0, 0.5, 2, 8, 24, and 72 h post-inoculation (hpi) using the RNA prep Pure Plant Kit (Tiangen Biotech, Beijing, China). cDNA synthesis was performed with the HiScript III RT SuperMix (Vazyme Biotech, Nanjing, China). Gene-specific primers for *VdPex30* and the reference gene *Vd-actin* (VDAG_00941) were designed using Primer 5 and validated for specificity. Reverse transcription quantitative PCR (RT-qPCR) was conducted using Taq Pro Universal SYBR qPCR Master Mix (Vazyme) on an Applied Biosystems 7500 Real-Time PCR System (Thermo Fisher Scientific, USA) under the following conditions: 95°C for 30 s, followed by 40 cycles of 95°C for 3 s and 60°C for 30 s (44). Relative expression levels were calculated using the 2^−ΔΔCt^ method (45). The amplification efficiency for each RT-qPCR primer set was determined by standard curve analysis and is provided in Table S2.

### HIGS assay

*Agrobacterium tumefaciens*-mediated transient gene silencing in cotton was conducted using a Tobacco rattle virus (TRV)-based HIGS system. A 400-bp fragment of *VdPex30* was amplified and cloned into the TRV2 vector using *Eco*R I and *Bam*H I restriction sites to generate TRV2::VdPex30. The recombinant plasmid, together with TRV1, TRV2::00 (empty vector control), and TRV2::*CLA1* (positive control), was introduced into *Agrobacterium tumefaciens* strain GV3101 (46). *Agrobacterium* cultures were grown overnight at 28°C in LB medium supplemented with rifampicin (50 μg/mL) and kanamycin (100 μg/mL), harvested by centrifugation (4,000 × *g* for 10 min), and resuspended in infiltration buffer containing 10 mM MgCl_2_, 10 mM MES (pH 5.6), and 200 μM acetosyringone. Cell suspensions were adjusted to OD_600_ = 0.8–1.0 and incubated at room temperature for 3 h in the dark. Equal volumes of *Agrobacterium* suspensions carrying TRV1- and TRV2-derived constructs were mixed at a 1:1 ratio prior to infiltration.

Two-week-old cotton (*Gossypium hirsutum* L. cv. Coker 312) seedlings at the cotyledon stage were used for agroinfiltration. The mixed *Agrobacterium* suspensions were infiltrated into both cotyledons using a needleless syringe. Plants infiltrated with TRV2::00 served as the negative control. TRV2::*CLA1* was used as a positive control for TRV-based silencing because silencing of *CLA1* causes a characteristic photobleaching phenotype, indicating successful systemic silencing (14).

After the photobleaching phenotype was clearly observed in TRV2::*CLA1*-inoculated plants, cotton seedlings infiltrated with TRV2::VdPex30 and TRV2::00 were sampled to evaluate the silencing efficiency of *VdPex30* by RT-qPCR. Primers used for RT-qPCR detection of VdPex30 were designed based on the *V. dahliae* genome sequence and showed no significant homology to cotton transcripts. Primer specificity was confirmed by melt curve analysis, indicating amplification of a single specific product. Only seedlings showing effective gene silencing were subsequently used for pathogenicity assays. Following silencing validation, cotton seedlings were inoculated with *V. dahliae* spore suspensions by root inoculation. Disease symptoms were assessed, and disease severity was evaluated at 14 dpi. At the same time point, root tissues were collected for genomic DNA extraction, and fungal biomass was quantified by RT-qPCR targeting the *V. dahliae* ITS region. Fungal biomass was quantified by RT-qPCR using the *V. dahliae*-specific ITS primer pair Vd-ITS-F/R, with the cotton Polyubiquitin gene used as the internal reference for normalization.

### Generation of *VdPex30*-RNAi transgenic tobacco

*VdPex30*-RNAi transgenic tobacco with stable inheritance was generated via Gateway technology (47). The *VdPex30* cDNA fragment (with BP sites) was amplified by two-step PCR using *VdPex30*-RNAi-F/R primers (attB1/attB2 adapters). Purified products were recombined with pDONR207 via BP reaction (Gateway BP Clonase II; 25°C for 1 h) and transformed into One Shot OmniMAX 2 T1 cells, with positives selected on gentamicin-LB and verified by attB-F/R primers. The BP plasmid was then subjected to LR reaction with pk7GWIWG2(I) vector (Gateway LR Clonase II), followed by selection on spectinomycin-LB. Positive clones were introduced into *Agrobacterium* LBA4404. Tobacco leaf discs (0.4 × 0.6 cm) were infected with bacterial suspension (OD_600_ = 0.1–0.2) for 5 min, co-cultured on shoot induction medium (MS + 0.2 mg/L NAA + 2 mg/L 6-BA; 25°C, dark, 3 days), and then transferred to selection medium (100 mg/L Kan + 500 mg/L Carb) under 16 h light/8 h dark for 14–21 days. Resistant shoots were rooted on MS (Kan + Carb), acclimatized, and transplanted to soil (nutrient soil:vermiculite = 1:1). T_1_ positives were confirmed by PCR (*VdPex30*-RNAi-test-F/R primers; Table S1).

### Gene knockout and complementation

The *VdPex30* strains were generated by transferring the knockout plasmid pGKO-*VdPex30* into protoplasts isolated from V991 using polyethylene glycol (PEG)-mediated transformation (48). Protoplasts of Δ*VdPex30* strains were similarly transformed with the complementary plasmid pCM-Hyg-*VdPex30* to obtain the complementary mutant strains (Δ*VdPex30*-C). Mutant strains were preliminarily selected using antibiotic stress and single-spore isolation and then confirmed by genomic PCR using specific primers (Table S1). Δ*VdPex30* strains were selected on PDA in the presence of geneticin (G418, 50 μg/mL) and confirmed by PCR with primers (Table S1). Δ*VdPex30*-C strains were cultured and selected on PDA plates containing hygromycin B (50 μg/mL). Primers Hyg-F/Hyg-R and Pex30-F/Pex30-R (Table S1) were used for genomic PCR to check whether the complementation was successful.

### Oxidative stress assay and detection of intracellular ROS levels

Oxidative stress was assessed using the method described in previous research. A conidial suspension (500 µL, 5 × 10^6^ spores/mL) of each strain was spread evenly on Czapek Dox plates (49). Then, 100 μL H_₂_O_₂_ (100 mM) was poured into a hole punched by a sterile cork borer (5 mm in diameter) in the center of the plate. Plates were incubated at 25°C, and the diameter of the inhibition zone was measured after 7 days. For each strain, five replicate plates were prepared, and the experiment was performed independently three times.

For intracellular reactive oxygen species detection, 2 μL of a spore suspension (1 × 10^4^ spores mL^−1^) was inoculated into 20 μL of CM liquid medium and incubated at 25°C for 3 days. ROS levels were then qualitatively assessed using a ROS Testing Kit (Genmed Scientific Inc., Shanghai, China) according to the manufacturer’s instructions. Green fluorescence was observed using a confocal microscope (LSM 980) with excitation at 488 nm and emission collected at 500–550 nm. Fluorescence images were acquired under identical imaging conditions. ROS-associated fluorescence intensity was quantified using ImageJ software (NIH, USA). Regions of interest were selected, and mean gray values were measured after background subtraction. At least three independent biological replicates were analyzed.

### Stress and carbon utilization assays

Vegetative growth of the *V. dahliae*, Δ*VdPex30*, and Δ*VdPex30*-C strains on different media was compared. Each strain was first cultured in complete medium and filtered through a sterile 40 μm Falcon Cell Strainer (New York, NY, USA) to collect conidia. A drop of a conidial suspension (10 μL, 2 × 10^6^ spores/mL) of the respective strains was placed in the center of a plate of Czapek Dox agar with sucrose (30 g/L) and without sucrose, pectin (10 g/L), xylose (10 g/L), starch (17 g/L), or galactose (10 g/L). A 10 μL drop of a conidial suspension (2 × 10^6^ spores/mL) was also placed in the center of PDA plates. For stress media assays, PDA-based stress media were prepared as follows: 75 μg/mL Congo red (CR) (Solarbio, Beijing, China) (3.75 mL of 2% stock solution per liter), 30 μg/mL Calcofluor white (CFW) (Coolaber, Beijing, China) (1.5 mL of 2% stock solution per liter), 0.01% sodium dodecyl sulfate (SDS) (10 mL of 1% stock solution per liter), 182,170 μg/mL sorbitol (SBT), and 40,950 μg/mL NaCl. The plates were incubated at 25°C in the dark. Each strain was tested on five plates of each medium type. Colony morphology was photographed, and diameters were measured after 14 days. The mean colony diameter of *V. dahliae* was compared with that of the Δ*VdPex30* and Δ*VdPex30*-C strains for each carbon source and stress condition tested. Stress tolerance assays were performed on potato dextrose agar medium supplemented with different stress-inducing agents. For each stress condition, PDA medium was supplemented individually with one of the following compounds: CR (75 μg mL^−1^), CFW (30 μg mL^−1^), SDS (0.01%, wt/vol), SBT (182.17 mg/mL), or sodium chloride (NaCl; 40.95 mg mL^−1^). Stock solutions were prepared at appropriate concentrations and added to autoclaved PDA medium after cooling to approximately 55°C prior to plate pouring.

### Transcriptome analysis/RNA-seq

To investigate transcriptional changes associated with gene deletion, RNA-seq was performed using the wild-type strain and the mutant strain cultured on potato dextrose agar medium. Mycelia were harvested for RNA extraction at the indicated time points. Total RNA was extracted using a fungal total RNA extraction kit according to the manufacturer’s instructions, and RNA integrity and quality were assessed using an Agilent 2100 Bioanalyzer.

RNA-seq libraries were prepared by enriching poly(A)+ RNA, followed by fragmentation and strand-specific cDNA synthesis. Subsequently, end repair, Illumina adaptor ligation, and PCR amplification were performed to generate sequencing libraries. After quality assessment, libraries were sequenced on an Illumina NovaSeq 6000 platform to obtain paired-end reads.

Raw sequencing data were subjected to quality control using FastQC, and adaptor sequences as well as low-quality reads were removed using Cutadapt. Clean reads were mapped to the reference genome using Bowtie2 (50). Gene-level read counts were generated using Subread (51), and differential expression analysis was conducted based on log_2_-transformed fold changes between the mutant and wild-type strains. Genes with |log_2_(fold change)| ≥ 1 and an adjusted *P*-value (FDR) < 0.05 were defined as differentially expressed genes (DEGs).

Functional annotation and enrichment analyses were performed using the ClusterProfiler package, including Gene Ontology (GO) enrichment and Kyoto Encyclopedia of Genes and Genomes (KEGG) pathway enrichment analysis (https://www.genome.jp/kegg/) (52).

### Y2H screening

To identify potential protein interaction partners of VdPex30, the full-length coding sequence of the *VdPex30* gene was amplified and cloned into the pGBKT7 vector (Coolaber, Beijing, China) to generate the bait construct. Meanwhile, a high-quality cDNA library of *V. dahliae* was constructed and ligated into the pGADT7 vector (Coolaber, Beijing, China), which served as the prey construct (53). Transformed yeast cells (Y2HGold strain) were initially selected on SD/-Trp/-Leu dropout medium to ensure the presence of both plasmids. For interaction screening, colonies were transferred onto more stringent quadruple dropout medium (SD/-Trp/-Leu/-His/-Ade) supplemented with X-α-Gal to assess the activation of reporter genes (*HIS3*, *ADE2*, and *MEL1*). Colonies exhibiting growth and blue coloration were considered positive for protein–protein interaction. Plasmids from positive yeast colonies were extracted and subjected to Sanger sequencing to identify the interacting cDNA inserts. The resulting sequences were annotated using the InterPro database (https://www.ebi.ac.uk/interpro/) to predict functional domains and protein families. In addition, interaction networks were inferred using the STRING database (https://string-db.org/), while potential metabolic or signaling pathways involving the candidate proteins were analyzed through the KEGG pathway database (https://www.genome.jp/kegg/).

### LUC assay

For the split-LUC assay (54), *A. tumefaciens* strains harboring pCAMBIA-VdPex30-nLUC and pCAMBIA-VdMIase-cLUC were co-infiltrated into *Nicotiana benthamiana* leaves, with each strain adjusted to OD_600_ = 0.5. Two days post-infiltration, the infiltrated leaf areas were treated with 1 mM luciferin (Solarbio, Beijing, China; Cat. D8390) and incubated in the dark for 5 min prior to CCD imaging using the IVIS Lumina LT system (PE, USA).

### BiFC assay

For the BiFC assay, transformants harboring VdPex30-YFP^N^ and VdMIase-YFP^C^ were initially streaked onto PDA medium using a sterile inoculating loop. The inoculated plates were incubated in a constant-temperature incubator at 25°C in the dark for 3 days to allow colony formation, as previously described (55). Subsequently, actively growing fungal mycelia from the edge of 3-day-old colonies were carefully harvested using a sterile scalpel and transferred onto glass slides. A drop of sterile distilled water was added to each slide to gently disperse the mycelia, followed by covering with a coverslip to avoid air bubbles, ensuring optimal visualization conditions for fluorescence microscopy. YFP signals were captured using a confocal microscope (LSM 980) with excitation at 514 nm, and the emission spectra were collected within the range of 524–550 nm. This assay was conducted with three independent biological replicates.

### Statistical analysis

Unless otherwise stated, all experiments were independently repeated three times, with at least three biological replicates per experiment (total *n* ≥ 9 per treatment). Data are presented as mean ± SD, calculated after confirming normality and equal variance. Students’ *t*-test (Excel) analyzed differences in colony/lesion diameter, conidiation, appressoria development, inhibition rate, and microsclerotia traits. Gene expression and biomass differences were assessed via one-way ANOVA with LSD *post hoc* test. Sugar effects on strains were analyzed by MANOVA. All statistics were performed using SPSS 19 (SPSS Inc., Chicago, IL, USA).

## RESULTS

### VdPex30 is a basic and hydrophilic peroxisomal protein with conserved domains and potential transmembrane features

**Fig 1:**
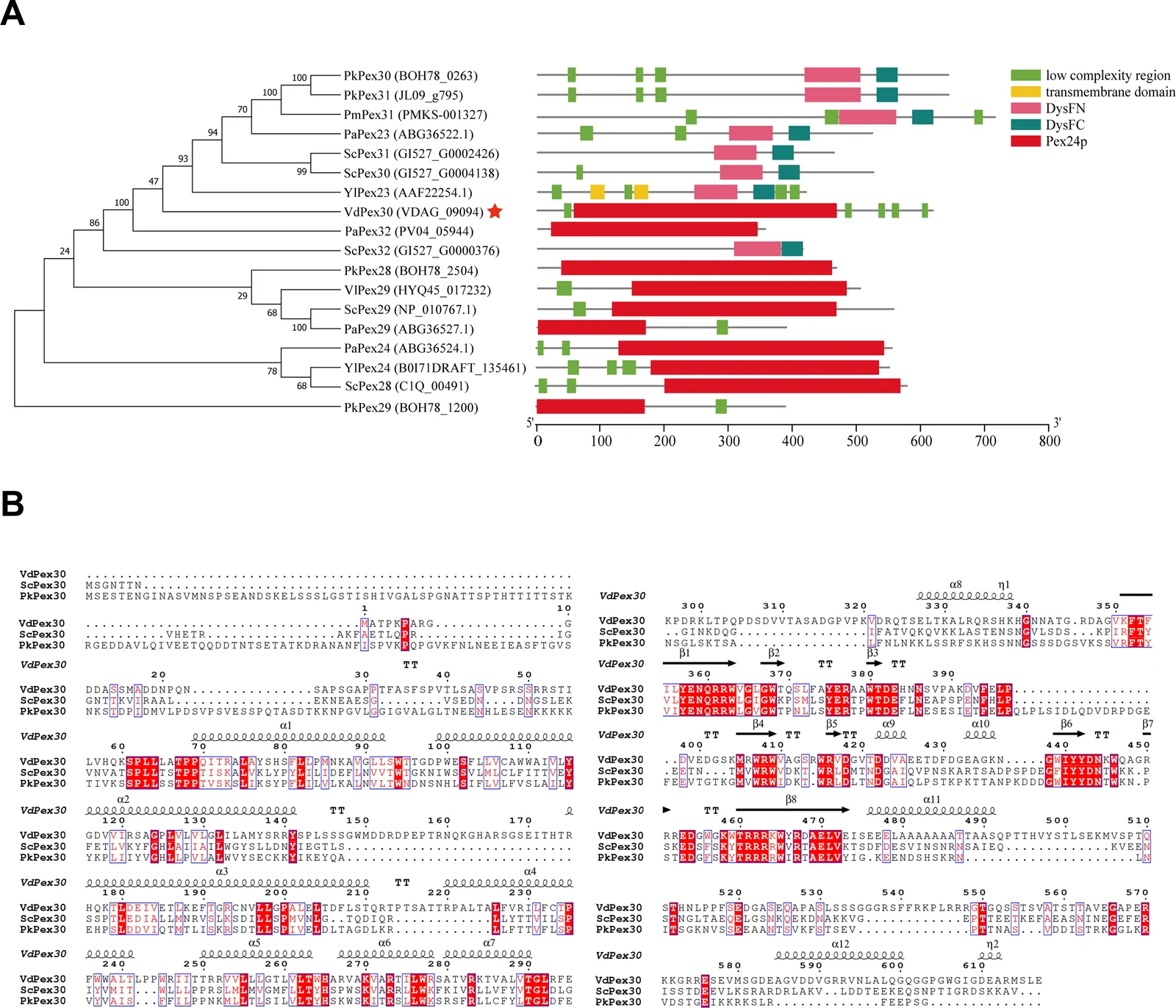
Sequence alignment and evolutionary analysis reveal structural conservation and domain features of the Pex30 protein family. (**A**) Phylogenetic tree and domain analysis of VdPex30 homologous proteins from different species. VdPex30 is highlighted with an asterisk, and modules of different colors represent different structural domains. The domains are plotted concerning the prediction results of the Pfam and SMART databases. (**B**) Multiple sequence alignment of VdPex30, ScPex30, and PkPex30. The common residues of the three sequences are displayed in white letters on a red square, and similar residues are shown in red. The black dot represents a single-amino-acid deletion, whereas the black arrow and coiled helix represent the predicted secondary structure.

An in-depth structural analysis of VdPex30 is essential for understanding its function. VdPex30 encodes a 671-residue peroxisomal membrane protein (68.07 kDa; C_₃₀₀₉_H_₄₇₂₉_N_₈₇₉_O_₉₀₄_S_₁₂_), containing the conserved Pex24p domain. It shows an instability index of 58.67, while an aliphatic index of 74.02 and a GRAVY value of –0.446 indicate hydrophilicity. Secondary structure prediction revealed that VdPex30 consists of 27.55% α-helices, 15.24% extended strands, and 54.46% random coils. Multiple sequence alignment showed conserved residues with Pex30 homologs from *Saccharomyces cerevisiae* and *Pichia kudriavzevii* (Fig. 1B). The three-dimensional structure of VdPex30 has a core domain rich in α-helices, with few β-sheets and abundant random coils (Fig. S1A). Phylogenetic analysis with Pex24p superfamily from *Verticillium*, *Saccharomyces*, and *Pichia* species revealed that VdPex30 clusters closely with YlPex23 from *Yarrowia lipolytica* (Fig. 1A). TMHMM transmembrane domain prediction results showed that two regions of VdPex30 (aa 123–129 and 227–239) had a high probability (>0.85), suggesting the potential existence of atypical membrane regions (Fig. S1B). These results suggest that VdPex30 is an evolutionarily conserved peroxisomal membrane protein with characteristic structural features and potential atypical transmembrane regions that may underlie its functional role.

### *VdPex30* is highly expressed during early infection and is a potential HIGS target against *V*. *dahliae*

**Fig 2:**
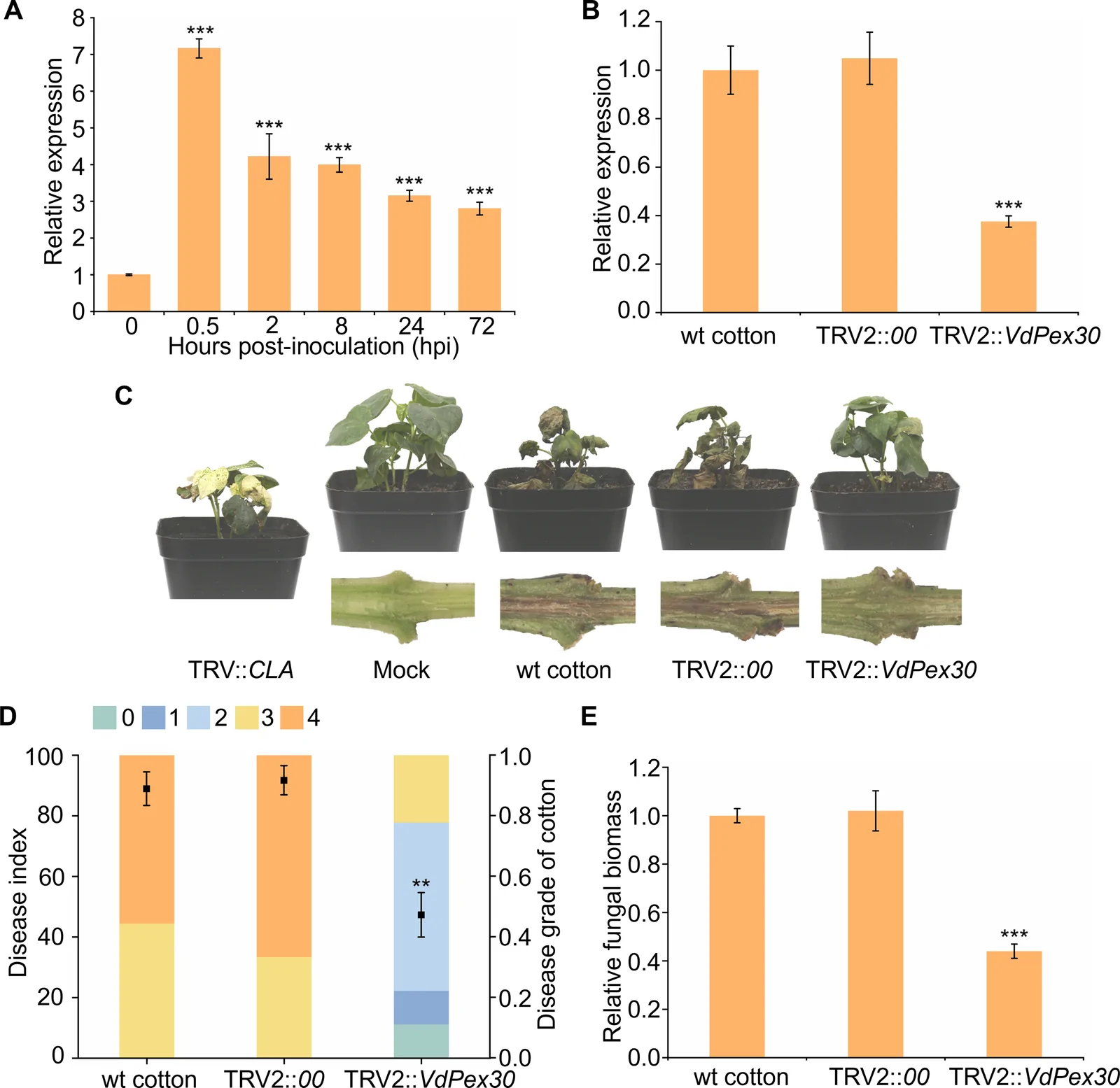
Impact of *VdPex30* silencing on fungal infection and plant disease development. (**A**) *VdPex30* expression in cotton after *V. dahliae* inoculation. Twenty-one-day-old cotton plants were inoculated with *V. dahliae* spore suspension (10^7^ cfu/mL) by root soaking, and samples were collected at 0, 0.5, 2, 8, 24, and 72 hpi for RT-qPCR analysis. (**B**) RT-qPCR detection of the expression level of *VdPex30*. Cotton plants were injected with *TRV1 + TRV2::VdPex30*, *TRV1 + TRV2::00* (empty vector control), and *TRV1 + TRV2::CLA1* (positive control) at the cotyledon stage. After the positive control plants exhibited the photobleaching phenotype, cotton plants were inoculated with *V. dahliae*. Root samples were collected at 14 dpi for RNA extraction and reverse transcription. The relative expression level of *VdPex30* was then determined by RT-qPCR. (**C**) Phenotype observation. (**D**) Disease index. (**E**) Fungal biomass of different treated cotton plants. The data were recorded at 14 dpi. Different colors represent different disease levels, and the proportion of color modules in the column represents the percentage of diseased plants with different disease levels in the total number of diseased plants. Disease severity was evaluated at 14 dpi and quantified using the 0–4 disease index scale described above. There were three independent samples for each treatment. **P < 0.01 and ***P < 0.001 (Student’s *t*-test).

To determine whether *VdPex30* plays a role in *V. dahliae* infection, we analyzed its expression pattern at 0, 0.5, 2, 8, 24, and 72 h post-inoculation using quantitative real-time polymerase chain reaction. The results showed that compared to 0 hpi, *VdPex30* expression rapidly increased approximately sixfold at 0.5 hpi and maintained a high level of performance up to 72 hpi. These findings indicate that *VdPex30* is highly expressed during the early stages of *V. dahliae* infection, implying its potential role in pathogenicity (Fig. 2A).

To further elucidate the function of *VdPex30*, a 400-bp fragment from its coding sequence was selected for HIGS. Pathogenicity of the *V. dahliae* on cotton was evaluated using the root-dip inoculation method at 14 days post-inoculation. RT-qPCR results showed that the transient silencing efficiency of *VdPex30* reached approximately 60% (Fig. 2B). Phenotypic assessment revealed that wild-type (wt) cotton and *TRV2::00* control groups displayed severe wilting, chlorosis, and even plant death, with extensive vascular browning (Fig. 2C). These groups exhibited significantly higher disease severity indices and fungal biomass in roots, indicating a high level of infection. In contrast, transient silencing of *VdPex30* led to significantly reduced leaf wilting, attenuated vascular discoloration, lower disease indices, and a substantial decrease in fungal biomass (Fig. 2D and E). These results suggest that disruption of *VdPex30* expression impairs fungal pathogenicity of *V. dahliae* and suppresses VW symptoms on cotton.

**Fig 3:**
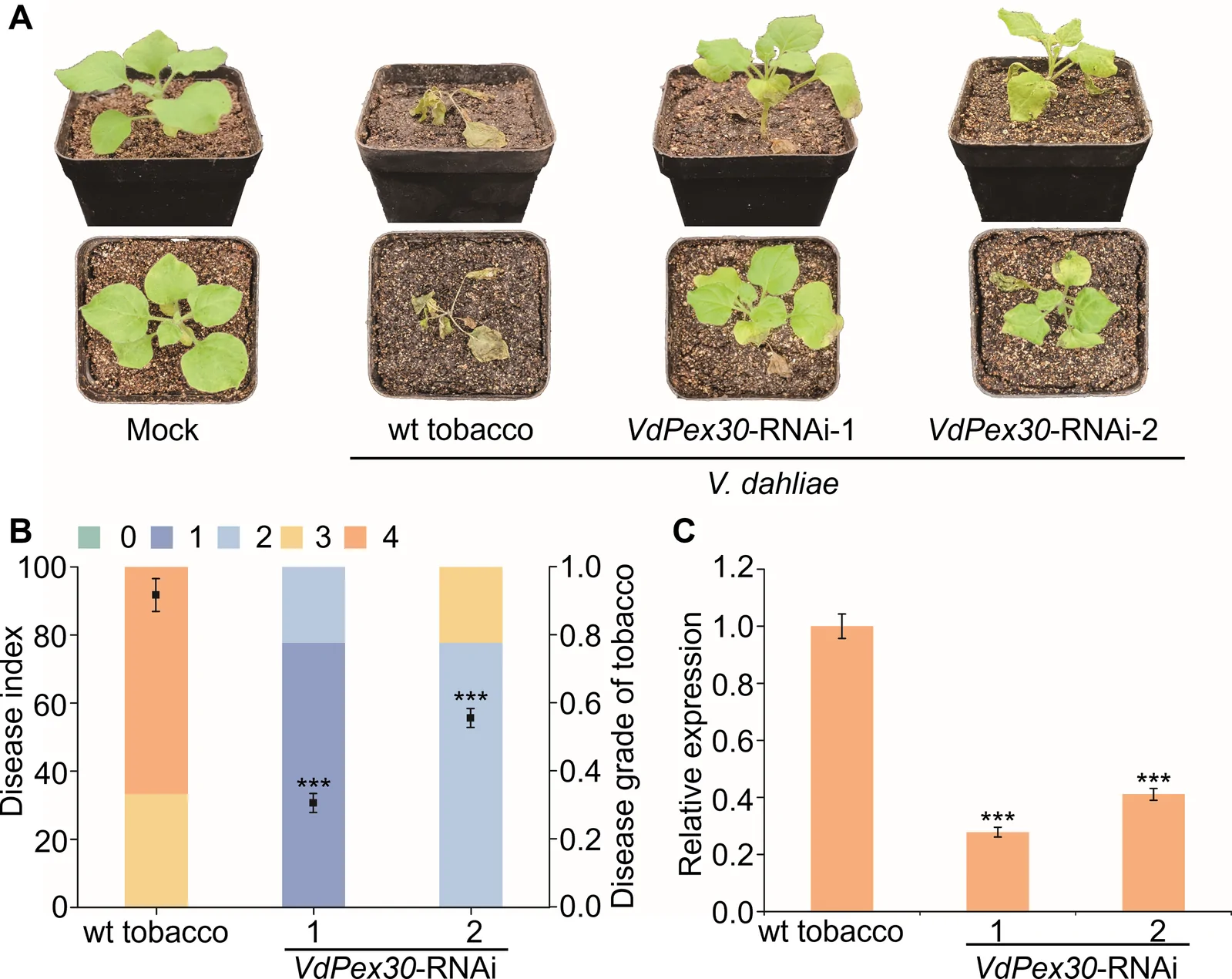
*VdPex30*-RNAi transgenic tobacco exhibits reduced susceptibility to VW. (**A**) The biological phenotype of *VdPex30*-RNAi transgenic tobacco infected with *V. dahliae*. T2 generation positive plants at the four to five leaf stage were infected with *V. dahliae* via root soaking (spore suspension concentration 10^7^ cfu/mL), observed at 14 dpi. (**B**) The disease index of *VdPex30*-RNAi transgenic tobacco. Different colors represent different disease levels, and the proportion of color modules in the column represents the percentage of disease plants with different disease levels in the total number of disease plants. (**C**) The expression level of *VdPex30* of *V. dahliae* from transgenic tobacco roots. The expression level of *VdPex30* in *V. dahliae* isolated from the roots of transgenic tobacco, detected by RT-qPCR at 14 dpi. There were three independent samples for each treatment. ***P < 0.001 (Student’s *t*-test).

To further explore the potential role in disease resistance, we generated *VdPex30*-RNAi transgenic tobacco plants (Fig. S2). This transgenic strategy allowed us to evaluate whether plant-mediated silencing of the fungal VdPex30 gene could enhance host resistance to *V. dahliae* infection. To evaluate disease resistance, T2 generation positive transgenic lines were inoculated with a spore suspension of *V. dahliae*. Control treatments included: (i) wt tobacco inoculated with the spore suspension as a positive control, and (ii) wt tobacco treated with sterile water (mock) as a negative control. As shown in Fig. 3A, wt tobacco plants exhibited severe wilting, collapse, and even death. In contrast, transgenic lines 1 and 2 displayed milder symptoms, including chlorosis and leaf desiccation. The disease severity in *VdPex30*-RNAi tobacco was significantly lower than that in wt tobacco, demonstrating that *VdPex30*-RNAi transgenic tobacco effectively enhances resistance to VW (Fig. 3B). The RT-qPCR analysis revealed that *VdPex30* expression levels were significantly reduced in *V. dahliae* infecting *VdPex30*-RNAi transgenic tobacco compared to those in wt tobacco (Fig. 3C). These results confirm that *VdPex30*-RNAi tobacco could efficiently silence *VdPex30* in *V. dahliae*, thereby conferring enhanced disease resistance.

### Deletion of *VdPex30* resulted in increased susceptibility to oxidative stress

To further investigate the comprehensive role of *VdPex30* in the pathogenic process, Δ*VdPex30* strains were generated through homologous recombination in the *V. dahliae* strain. Subsequently, ectopic complemented strains (Δ*VdPex30-C*) were constructed by reintroducing *VdPex30* into Δ*VdPex30* strains. All mutant strains were verified by multiple diagnostic PCR assays (Fig. S3A through H).

**Fig 4:**
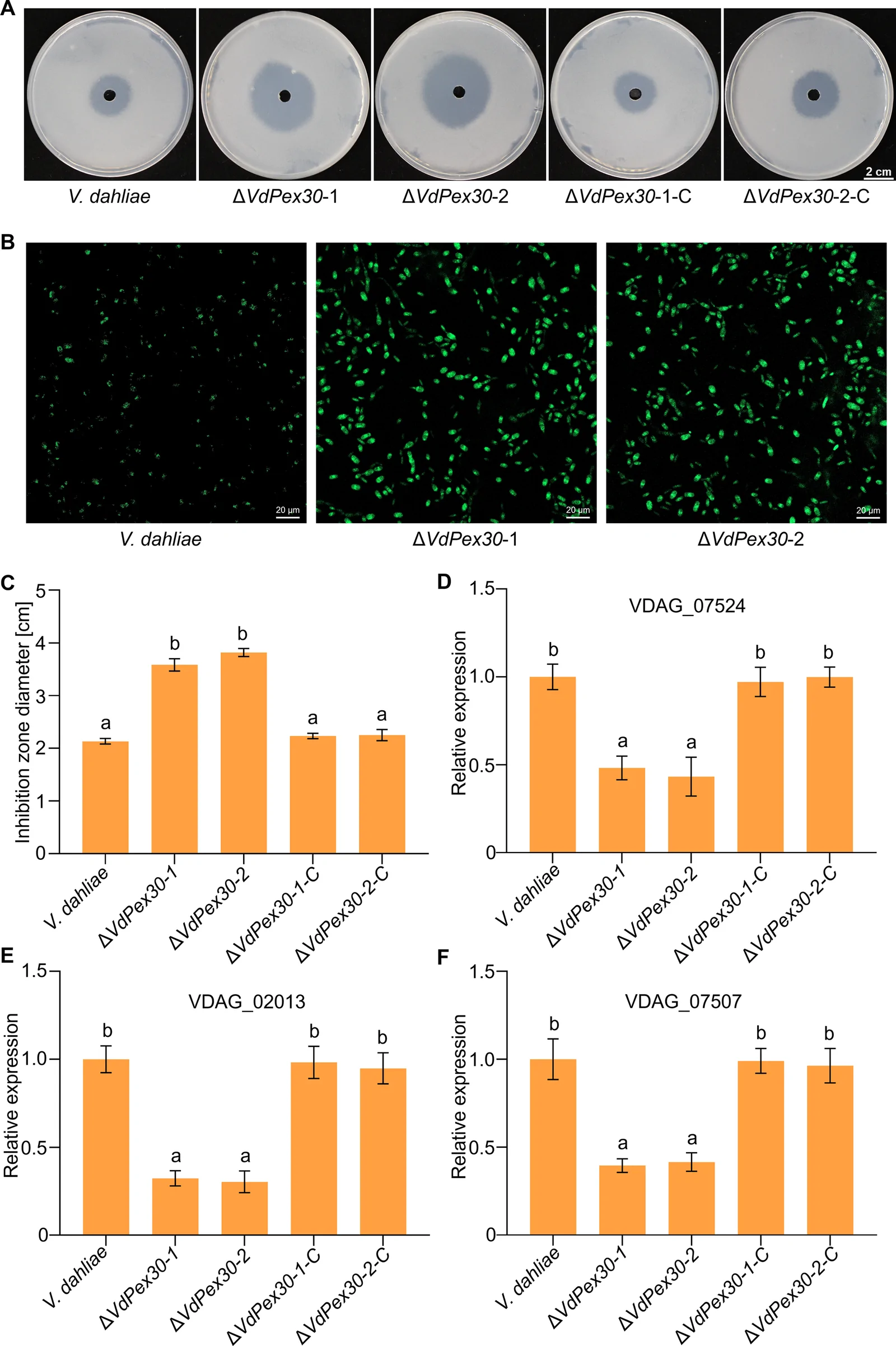
*VdPex30* mediates oxidative stress response in *V. dahliae*. (**A**) Inhibition zones after 1 week in the oxidative stress assay. For each strain, 100 μL was spread evenly on the Czapek Dox plates. One hundred microliters of H_2_O_2_ (100 mM) was poured into a hole in the center of the plate. (**B**) ROS levels in hyphae from 3-day-old CM culture. Hyphae were stained with the ROS Testing Kit and viewed with confocal microscopy. Bars: 20 μm. (**C**) Mean (±SD) diameter of the H_2_O_2_ inhibition zone. Three independent replicates were completed. Different letters above the bars indicate a significant difference among treatment groups (*P* < 0.01). Bars labeled with different letters are significantly different, whereas bars sharing the same letter are not significantly different, as determined by one-way ANOVA followed by Tukey’s HSD test. (**D–F**) Mean (±SD) gene relative expression analysis. Conidia harvested from the plates were cultured in CM for RNA extraction. RT-qPCR reactions were performed using specific primers. The experiment was performed with three independent biological replicates. Letters above bars indicate significant differences among strains as described in panel **C** (*P* < 0.01). The expression levels of (**D**) glutathione reductase (*VdGR*, VDAG_07524), (**E**) NADPH oxidase A (*VdNoxA*, VDAG_06812), and (**F**) superoxide dismutase (*VdSOD5*, VDAG_07507).

Given that VdPex30 is a peroxisomal membrane protein and may play a role in ROS metabolism, we hypothesized that the increased oxidative stress sensitivity observed in Δ*VdPex30* was associated with impaired ROS homeostasis*.* To test this hypothesis, we evaluated the sensitivity of each strain to H_₂_O_₂_ exposure by measuring the diameter of the inhibition zone. The zone of inhibition for Δ*VdPex30*-1 and Δ*VdPex30*-2 strains was larger than the wt and complemented strains (Fig. 4A and C). We further measured intracellular ROS levels of each strain. As expected, Δ*VdPex30* showed brighter green fluorescence (Fig. 4B), indicating higher intracellular ROS levels compared to *V. dahliae*. Semi-quantitative analysis further supported these observations (Fig. S5). This suggests that the deletion of *VdPex30* leads to increased ROS accumulation. To elucidate the molecular basis of this phenotype, we examined the expression of key genes involved in the ROS pathway. The deletion of *VdPex30* resulted in downregulated expression of glutathione reductase (*VdGR*, VDAG_07524), NADPH oxidase A (*VdNoxA*, VDAG_06812), and superoxide dismutase (*VdSOD5*, VDAG_07507) (Fig. 4D through F). Collectively, these findings suggest that the deletion of *VdPex30* increases sensitivity to H_₂_O_₂_ and promotes intracellular ROS accumulation, likely due to the downregulation of genes associated with oxidative stress responses.

### *VdPex30* is required for carbon source utilization, stress adaptation, and full virulence in *V. dahliae*

**Fig 5:**
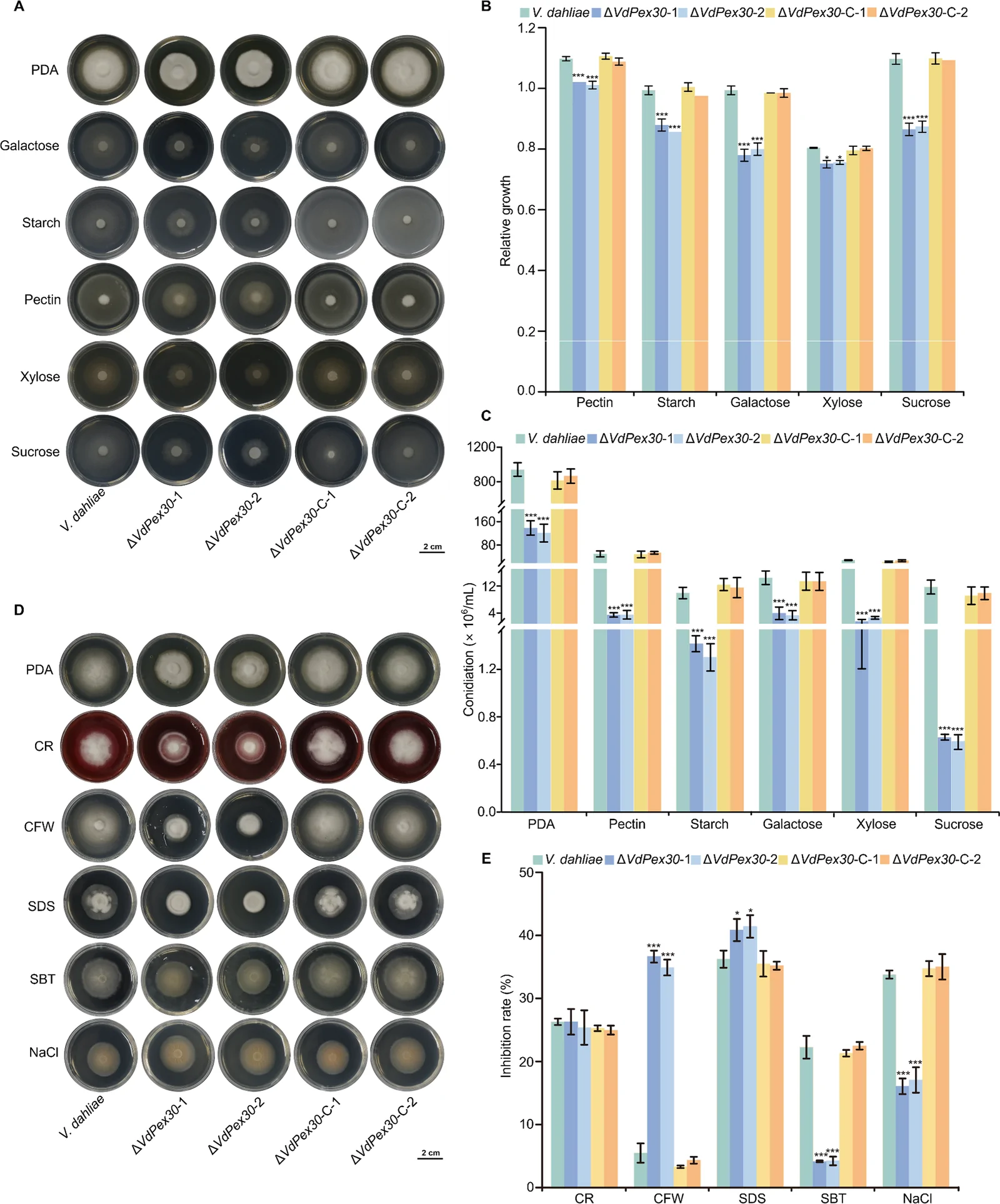
Role of *VdPex30* in carbon metabolism and stress adaptation in *V. dahliae*. (**A**) The growth phenotypes of *V. dahliae*, Δ*VdPex30,* and Δ*VdPex30*-C in different carbon sources. The growth phenotypes of *V. dahliae*, Δ*VdPex30,* and Δ*VdPex30-C* were cultured on Czapek medium supplemented with different carbon sources (30 g/L sucrose, 17 g/L starch, 10 g/L pectin, 10 g/L xylose, and 10 g/L galactose) at 25°C for 14 days. *V. dahliae* was inoculated on medium containing kanamycin and carbendazim (Car), while Δ*VdPex30* and Δ*VdPex30-C* were inoculated on medium containing hygromycin, Kan, and Car, with 10 μL spore suspension (10^6^ cfu/mL) per plate. (**B**) The relative growth diameter of *V. dahliae*, Δ*VdPex30*, and Δ*VdPex30*-C in different carbon sources. (**C**) The sporulation capacity of *V. dahliae*, Δ*VdPex30,* and Δ*VdPex30*-C in different carbon sources. (**D**) The growth phenotype of *V. dahliae*, Δ*VdPex30,* and Δ*VdPex30*-C in different stress conditions. PDA was supplemented with 75 μg/mL CR, 30 μg/mL CFW, 0.01% SDS, SBT (182,170 μg/mL), or NaCl (40,950 μg/mL). Inoculation conditions were consistent with panel **A**, and cultures were incubated at 25°C for 14 days. (**E**) The growth diameter inhibition rate of *V. dahliae*, Δ*VdPex30,* and Δ*VdPex30*-C in different stress conditions. There were three independent samples for each treatment. *P < 0.05 and ***P < 0.001 (Student’s *t*-test).

To further investigate the role of *VdPex30*, we cultured *V. dahliae*, Δ*VdPex30*, and Δ*VdPex30-C* strains on media containing different carbon sources and under adverse conditions (Fig. 5A). On PDA medium, Δ*VdPex30* exhibited no obvious changes in colony morphology; however, its colony diameter and conidiation were significantly reduced compared to *V. dahliae*. The Δ*VdPex30*-C restored these phenotypic defects, indicating that *VdPex30* is essential for normal vegetative growth and sporulation in *V. dahliae*. Relative growth rates on different carbon sources, normalized to PDA medium, revealed that Δ*VdPex30* displayed significantly reduced growth on pectin, starch, galactose, and sucrose, whereas the difference was minimal on xylose (Fig. 5B). Furthermore, Δ*VdPex30* showed a marked decline in sporulation, while Δ*VdPex30*-C exhibited a phenotype similar to *V. dahliae*. These findings suggest that *VdPex30* regulates carbon source utilization, particularly in the breakdown of pectin, starch, galactose, and sucrose (Fig. 5C).

To explore whether *VdPex30* is involved in stress responses, we cultured the strains on PDA medium supplemented with different stress inducers, including CR (a fungal cell wall synthesis inhibitor), CFW (a fungal cell wall synthesis inhibitor), SDS (a cell membrane stress inducer), SBT (osmotic stress), and NaCl (high salinity). Fresh conidial suspensions were spotted onto these stress-containing media, and colony growth was assessed after 14 days (Fig. 5D). The results indicated that all strains exhibited growth inhibition under stress conditions, with *V. dahliae* and Δ*VdPex30*-C showing similar inhibition rates. Δ*VdPex30* displayed no significant difference in inhibition on CR medium compared to *V. dahliae* and Δ*VdPex30*-C. However, under CFW and SDS stress conditions, Δ*VdPex30* exhibited significantly higher sensitivity, while it showed increased tolerance under SBT- and NaCl-induced stress (Fig. 5E). Therefore, these results demonstrate that *VdPex30* plays a crucial role in regulating carbon source utilization and stress responses in *V. dahliae*.

### *VdPex30* is required for full pathogenicity of *V. dahliae* on cotton

**Fig 6:**
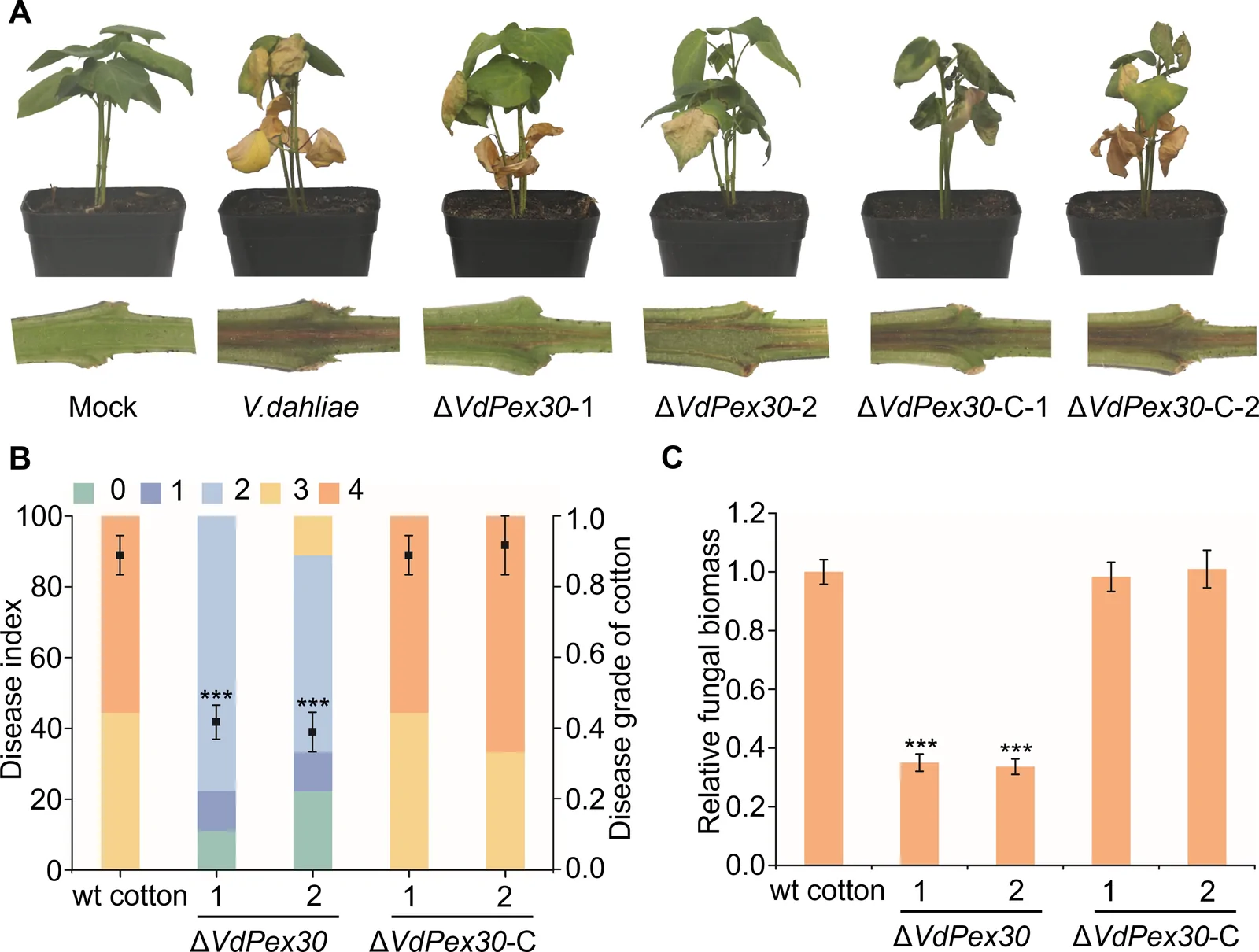
Pathogenicity detection of wt cotton, Δ*VdPex30*, and Δ*VdPex30*-C on cotton. (**A**) Pathogenic phenotype of wt cotton, Δ*VdPex30*, and Δ*VdPex30*-C on cotton. Cotton plants were inoculated with *V. dahliae*, Δ*VdPex30*, and Δ*VdPex30-C* via root dipping, with water treatment as a control. Phenotypes were observed at 14 dpi. (**B**) Disease index of *V. dahliae*, Δ*VdPex30*, and Δ*VdPex30*-C on cotton. Different colors represent different disease levels, and the proportion of color modules in the column represents the percentage of diseased plants with different disease levels in the total number of diseased plants. (**C**) Fungal biomass of *V. dahliae*, Δ*VdPex30*, and Δ*VdPex30*-C in cotton, determined by RT-qPCR at 14 dpi. DNA was extracted from mixed samples of 2 cm stem segments (above cotyledons) and root tissues, with Vd-ITS-F/R as detection primers (Table S1) and cotton polyubiquitin gene (Ubq, LOC107935373) as the internal reference gene. There were three independent samples for each treatment. ***P < 0.001 (Student’s *t*-test).

We conducted pathogenicity assays by inoculating cotton plants with spore suspensions of *V. dahliae*, Δ*VdPex30*, and Δ*VdPex30-C* strains, alongside a water treatment as a control for root immersion. The pathogenicity of the different strains was assessed by phenotypic observation and fungal biomass detection. Cotton plants inoculated with the wild-type *V. dahliae* strain V991 exhibited the most severe symptoms, including pronounced leaf wilting, yellowing, and extensive defoliation, with the entire plant exhibiting signs of wilting and even necrosis. In contrast, cotton plants inoculated with Δ*VdPex30* showed significantly reduced wilting and necrosis, with only mild yellowing observed on a few leaves (Fig. 6A). Upon reintroducing *VdPex30* into the Δ*VdPex30* strains, the disease symptoms in cotton plants were restored to the wt level. Quantitative analysis of disease index corroborated the phenotypic observations (Fig. 6B). Furthermore, we assessed fungal biomass at the roots of infected plants and found that the absence of *VdPex30* significantly reduced the pathogen’s colonization of cotton (Fig. 6C). In conclusion, *VdPex30* functions as a positive regulator contributing to the pathogenicity of *V. dahliae*.

### Transcriptomic profiling reveals that *VdPex30* modulates key pathways in carbon metabolism, stress adaptation, and pathogenicity in *V. dahliae*

**Fig 7:**
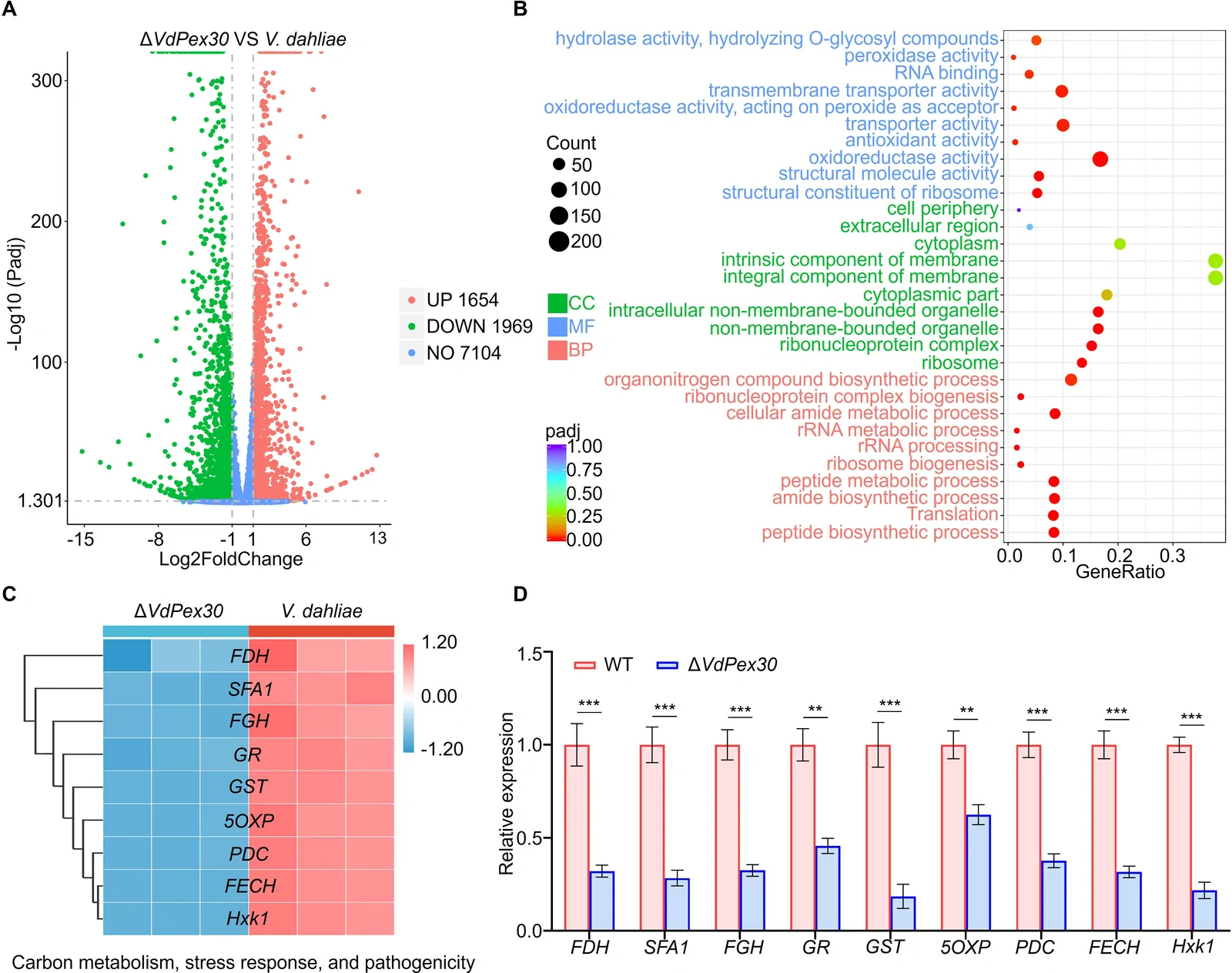
RNA-seq analysis reveals altered transcription in the Δ*VdPex30* strains of *V. dahliae*. (**A**) Visualization of DEGs using a volcano plot. (**B**) Bubble chart of KEGG enrichment analysis for DEGs. (**C**) Heatmap of DEGs associated with carbon metabolism, stress response, and pathogenicity, including *FDH* (VDAG_01315), *SFA1* (VDAG_00207), *FGH* (VDAG_06452), *GR* (VDAG_07524), *GST* (VDAG_02087), *5OXP* (VDAG_00175), *PDC* (VDAG_09443), *FECH* (VDAG_10248), and *Hxk1* (VDAG_05572), showed significant differential expression. (**D**) RT-qPCR validation of the target genes. The relative mRNA level of each gene was normalized to that of the *V. dahliae* actin gene. Data represent mean ± SD of three independent biological replicates. **P < 0.01 and ***P < 0.001 (Student’s *t*-test).

A comprehensive transcriptomic analysis was conducted to elucidate the molecular mechanisms underlying the role of *VdPex30* in *V. dahliae*. In total, 3,623 DEGs were identified between Δ*VdPex30* and *V. dahliae*, comprising 1,969 downregulated and 1,654 upregulated genes, while 7,104 genes exhibited no significant differential expression (Fig. 7A). The representative genes discussed below were selected based on their significant differential expression (|log2 fold change| ≥ 1, FDR < 0.05) and their involvement in major functional categories identified by GO and KEGG enrichment analyses. Focusing on carbon metabolism, Kyoto Encyclopedia of Genes and Genomes enrichment analysis highlighted significant involvement of *VdPex30* in central and specialized metabolic pathways (Fig. 7B). Notably enriched pathways included “pyruvate metabolism,” “starch and sucrose metabolism,” “glyoxylate and dicarboxylate metabolism,” and “pentose and glucuronate interconversions,” which indicates a vital role for *VdPex30* in dynamic carbon flux regulation and energy production, particularly under stress or nutrient-limited conditions. Importantly, the enrichment of carbon metabolism-related pathways in the RNA-seq analysis provides a molecular explanation for the pronounced growth defects of the Δ*VdPex30* strains on various carbon sources observed in the phenotypic assays.

Several pathways were enriched that are commonly associated with oxidative stress adaptation. “Nicotinate and nicotinamide metabolism” suggests a role for *VdPex30* in regulating NAD^+^/NADP^+^ homeostasis, which is crucial for antioxidant defense. Additionally, enrichment in “sulfur metabolism” and amino acid-related pathways, such as “cysteine and methionine metabolism,” implies a contribution to oxidative stress response through the regulation of thiol-containing compounds and sulfur assimilation. Additionally, pathways related to pathogenicity were also significantly represented. The most gene-rich pathway, “Biosynthesis of secondary metabolites,” suggests that *VdPex30* may influence the production of compounds involved in environmental adaptation and fungal virulence. Moreover, enrichment of transport and transcription-related pathways, such as “ABC transporters” and “RNA polymerase,” implies that *VdPex30* may regulate gene expression and molecular transport mechanisms essential for host-pathogen interactions.

All of the selected DEGs with these annotations were significantly downregulated in the Δ*VdPex30* samples, which are shown in Fig. 7C. They included genes related to fundamental roles in carbon metabolism, such as formate dehydrogenase (*FDH*), S-(hydroxymethyl)glutathione dehydrogenase (*SFA1*), and formylglutathione hydrolase (*FGH*). Additionally, key genes involved in stress response and pathogenicity were represented, including the genes encoding glutathione reductase (*GR*), glutathione S-transferase (*GST*), 5-oxoprolinase (*5OXP*), pyruvate decarboxylase (*PDC*), ferrochelatase (*FECH*), and hexokinase-1 (*HXK1*). The downregulation of these genes was verified by RT-qPCR, and the obtained results were consistent with the RNA-seq data (Fig. 7D). The reduced expression of genes related to carbon metabolism, stress response, and pathogenicity was observed in the Δ*VdPex30* strains and was associated with the reduced virulence phenotype of this strain. These transcriptomic changes further support the involvement of *VdPex30* in metabolic regulation, stress response, and pathogenicity in *V. dahliae*.

### Identification and validation of VdMIase as a VdPex30-interacting protein in *V. dahliae*

**Fig 8:**
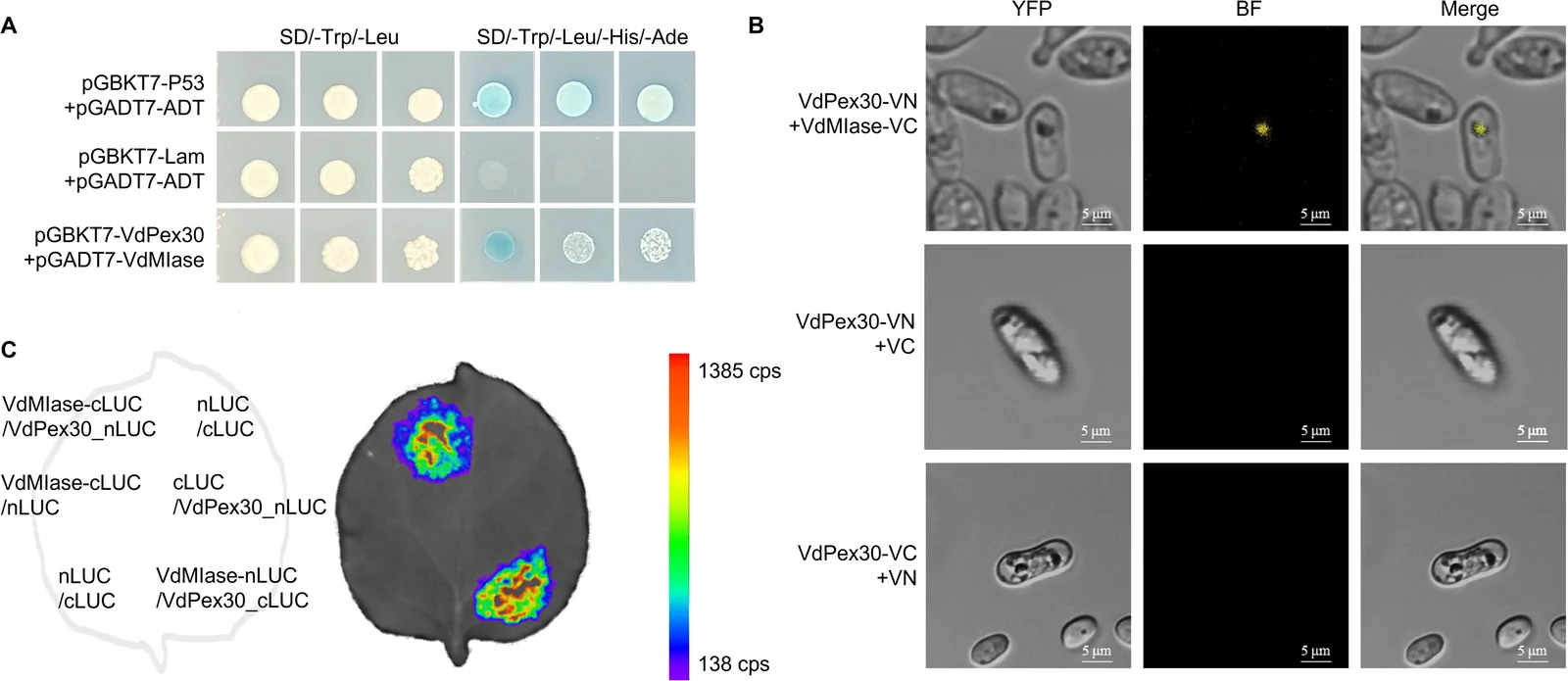
VdPex30 protein interactions and functional analysis. (**A**) Point-to-point verification of the interaction between VdPex30 and the candidate protein. Co-transformed yeasts were cultured on SD/-Trp/-Leu and SD/-Trp/-Leu/-His/-Ade/X-α-Gal, and the dilution ratios of yeast displayed in each column from left to right are 1/10, 1/100, and 1/1,000, respectively. (**B**) BiFC validates protein interaction in *V. dahliae*. Reconstituted YFP signals were observed under a confocal microscope, indicating direct protein–protein interaction *in vivo*. (**C**) Complementary imaging validation of VdPex30 with interacting proteins for firefly luciferase. Luciferase signal reconstitution reflects the physical association of the tested protein pairs.

To further elucidate the mechanism by which VdPex30 influences the pathogenicity of VW, we conducted a Y2H screen using the mating method to identify proteins interacting with VdPex30 in *V. dahliae*. Prior to the screening, control experiments confirmed that the pGBKT7-VdPex30 construct did not exhibit autonomous activation or toxicity in yeast (Fig. S4). This approach identified VDAG_01341 as a candidate protein potentially involved in the VdPex30-mediated regulatory network. Based on its predicted functional annotation, we designated this protein VdMIase (VDAG_01341) (Fig. 8A). BiFC assays confirmed the interaction between VdPex30 and VdMIase in *V. dahliae*, as evidenced by the specific restoration of fluorescence signals upon their co-expression (Fig. 8B). Furthermore, LUC assays demonstrated that the interaction between VdPex30 and VdMIase was specific (Fig. 8C). Collectively, these results demonstrate that VdPex30 physically interacts with VdMIase in *V. dahliae*.

## DISCUSSION

In recent years, peroxisome-associated proteins, particularly members of the Pex family, have attracted increasing attention due to their critical roles in fungal development and pathogenicity (56, 57). Studies in several plant pathogenic fungi, such as *M. oryzae*, *F. graminearum*, and *C. orbiculare*, have demonstrated that Pex proteins are indispensable for peroxisome biogenesis, lipid metabolism, stress adaptation, and virulence (33, 37, 38, 58–60). However, despite the economic importance of *V. dahliae* as a vascular wilt pathogen, the biological functions of Pex family proteins in this fungus remain largely unexplored (4, 40). In this study, we systematically characterized the biological function of the peroxisomal membrane protein VdPex30 in *V. dahliae*. Our results demonstrate that VdPex30 is essential for vegetative growth, carbon source utilization, stress tolerance, and full virulence. Transcriptomic and cellular analyses further reveal that VdPex30 coordinates metabolic homeostasis and oxidative stress responses during host infection. Notably, HIGS-mediated silencing of *VdPex30* significantly enhanced host resistance, underscoring its dual significance in fungal pathogenicity and as a potential target for RNAi-based disease control.

VdPex30 shares conserved functions with Pex30 family proteins across filamentous fungi (32). Peroxisomal membrane proteins are generally conserved across filamentous fungi and play important roles in peroxisome biogenesis and function (56). *Pex* genes have been shown to contribute to pathogenicity in plant pathogenic fungi; for example, deletion of *FAM1*, which contains a predicted Pex4-binding site, impairs fatty acid metabolism and virulence in *C. orbiculare* (60). In *Alternaria alternata*, deletion of the *AaPex3* disrupts peroxisome biogenesis, impairing fatty acid β-oxidation and leading to significant reductions in fungal growth, conidiation, and pathogenicity (61). In *Y. lipolytica*, *YlPex24* deletion disrupts peroxisome assembly by impairing the targeting of matrix and membrane proteins (62). In *S. cerevisiae*, ScPex28 and ScPex29 regulate peroxisome size, number, and distribution (63). The closest homolog, YlPex23, is essential for peroxisome biogenesis (64). Notably, VdPex30 shows high homology with YlPex24, ScPex28, and ScPex29 (Fig. 1A), suggesting conserved peroxisomal roles in *V. dahliae*.

Beyond its conserved role in peroxisome function, VdPex30 is also dynamically regulated during host infection and represents a promising target for disease control. During rice infection, the transcription factors *MOEITF1* and *MOEITF2* in *M. oryzae* are markedly upregulated at early stages, peaking at 12 hpi and suggesting involvement in early host interactions (65). Similarly, in *V. dahliae*, several *VdRGS* genes associated with signal transduction are induced early during infection, with *VdRGS1* showing the most prominent upregulation in cotton roots, indicating its key regulatory role in virulence (66). Consistent with these examples, *VdPex30* expression peaks during early infection (sixfold induction at 0.5 hpi) in this study (Fig. 2A). Combined with HIGS data in tobacco (17) and cotton, *VdPex30* emerges as a promising candidate for functional characterization. Critically, stable *VdPex30*-RNAi transgenic *Nicotiana* plants exhibited enhanced resistance (Fig. 3A through C), laying a foundation for RNAi-based biocontrol strategies potentially extendable to cotton.

Stress tolerance, particularly the ability to cope with host-derived oxidative stress, is a critical determinant of fungal pathogenicity. ROS are ubiquitous signaling molecules that play a pivotal role in plant defense responses (67). Upon pathogen invasion, plants often trigger excessive ROS accumulation, which induces localized cell death to restrict the spread of the pathogen (68). Therefore, the ability of phytopathogenic fungi to tolerate ROS is crucial for their growth and virulence (69). In *Candida glabrata*, the NADPH oxidase gene *CgNOX1* is essential for ROS generation. Deletion of *CgNOX1* impairs the oxidative stress response and reduces virulence (70). Similarly, in *M. oryzae*, the NADPH oxidase genes *MoNox1* and *MoNox2* regulate ROS production and pathogenicity (70). The cytotoxicity of excessive ROS contributes to the attenuated virulence observed in mutants with impaired ROS tolerance (71). Our oxidative stress assays revealed that Δ*VdPex30* strains exhibited significantly larger inhibition zones under H_₂_O_₂_ and accumulated higher intracellular ROS (Fig. 4A through C; Fig. S5), indicating enhanced oxidative stress sensitivity.

Plant cell walls, rich in carbohydrates, serve as primary carbon sources for pathogens (72, 73). Peroxisomes serve as key organelles in carbon metabolism for spore germination, infection structure formation, and pathogenicity (57, 74). In this context, Δ*VdPex30* showed impaired growth on sucrose, starch, pectin, xylan, and galactose, indicating defective carbon source utilization (Fig. 5A through C). Given that the degradation of plant cell walls is a critical factor for successful infection, these findings suggest that VdPex30 is essential for the efficient breakdown of plant cell wall components, thereby facilitating pathogenicity.

To successfully colonize their hosts, pathogens must bolster their resistance to a variety of host-derived stresses (75). In *Ustilaginoidea virens*, deletion of the *bZIP* transcription factor gene *UvbZIP6* resulted in diminished tolerance to oxidative, high-salt, and high-osmotic stresses. Conversely, sensitivity to cell wall stressors, including CFW and CR, was significantly heightened, thereby highlighting UvbZIP6’s role as a positive regulator of the cell wall stress response (76). Moreover, the growth of Δ*VdPex30* was significantly curtailed on media containing SBT and NaCl. These observations collectively imply that *VdPex30* functions as a positive regulator of tolerance to osmotic and salt stresses, as well as cell wall stress response (Fig. 5D through E). The distinct responses to different types of stressors further suggest that *VdPex30* may mediate stress responses through multiple pathways.

In *F. graminearum*, deletion of the peroxisomal genes *FgPEX1* and *FgPEX10* resulted in mutant strains (Δ*PEX1* and Δ*PEX10*) exhibiting reduced mycelial growth, decreased conidiation, and impaired formation of perithecia (59). The gene *FgPEX22*-like was found to be essential for both sexual and asexual reproduction. Deletion of *FgPEX22*-like led to significantly reduced fatty acid utilization, decreased production of the mycotoxin deoxynivalenol, and markedly diminished virulence. Moreover, the Δ*PEX22*-like mutants showed increased lipid droplet accumulation and impaired ROS scavenging capacity (37). The lack of *MoPex19* led to various defects in fungal development and pathogenicity in *M. oryzae* (58). Consistent with these findings, Δ*VdPex30* exhibited significantly reduced virulence on cotton (Fig. 6A through C), fully restored in Δ*VdPex30-*C, confirming *VdPex30*’s involvement in pathogenicity.

Transcriptome analysis of Δ*VdPex30* identified 3,623 DEGs (Fig. 7A). Key downregulated genes included: *SFA1*, which encodes S-(hydroxymethyl) glutathione dehydrogenase and is essential for nitric oxide detoxification, oxidative stress resistance, and biotrophy in *M. oryzae* (77); *GR*, which maintains redox homeostasis and sulfur stress tolerance in *Fusarium oxysporum* (78); *5OXP*, which regulates the glutathione cycle and virulence in *F. graminearum* (79); and *HXK1*, which functions in glucose metabolism and virulence regulation in *C. albicans* (80) (Fig. 7C). Ribosomes are central to protein translation, and dysfunction in their biogenesis can lead to impaired fungal growth and reduced pathogenicity (81). In addition, the pathway “Biosynthesis of secondary metabolites” indicates that *VdPex30* may regulate secondary metabolism related to environmental adaptation and virulence. Previous studies have shown that fungal secondary metabolites are involved in modulating host-pathogen interactions and suppressing host immune responses (82). Enrichment in “ABC transporters” and “RNA polymerase” pathways further supports a role for *VdPex30* in membrane transport and transcriptional regulation, essential for maintaining intracellular homeostasis and regulating gene expression during pathogenesis (83) (Fig. 7B). *VdPex30* also appears to participate in core carbon and nitrogen metabolic pathways, as evidenced by enrichment in “pyruvate metabolism,” “nitrogen metabolism,” and “alanine, aspartate, and glutamate metabolism.” These pathways are crucial for energy acquisition and nutritional adaptability during infection (84).

Nicotinic acid (niacin), via its enzymatically derived product NADPH, enables key antioxidant enzymes—including *VdGR*, *VdNoxA*, and *VdSOD5*—in generating and sustaining reduced antioxidants. These reduced antioxidants neutralize ROS such as O_₂_⁻ and H_₂_O_₂_, thereby maintaining cellular antioxidant systems (85, 86). Notably, MIase participates in the niacin metabolic pathway. In *Eubacterium barkeri*, niacin undergoes MIase-mediated isomerization during fermentation to pyruvate and propionate (87). Impaired niacin metabolism could restrict NAD(P)^+^ biosynthesis, disrupting core metabolism and weakening oxidative stress tolerance (88). In this study, we demonstrate that VdPex30 interacts with VdMIase (Fig. 8A through C). Thus, we speculate that the absence of *VdPex30* disrupts niacin metabolism, thereby downregulating the expression of *VdGR*, *VdNoxA,* and *VdSOD5*—genes integral to ROS homeostasis. This dysregulation elevates sensitivity to H_₂_O_₂_ and promotes intracellular ROS accumulation. Future studies will elucidate the regulatory networks linking niacin metabolism and antioxidant systems via the VdPex30-MIase interaction in *V. dahliae*. Compared with previous studies on Pex proteins that have largely focused on peroxisome biogenesis, lipid metabolism, or organelle dynamics in model fungi, this study uncovers a distinct role of Pex30 in coordinating carbon metabolism, stress responses, and pathogenicity in the vascular wilt pathogen *V. dahliae*. By integrating phenotypic characterization, transcriptomic profiling, and host-induced gene silencing, our work extends the functional scope of Pex proteins from intracellular metabolic regulation to direct involvement in host–pathogen interactions. Notably, the identification of VdMIase as a VdPex30-interacting partner further provides a mechanistic link between peroxisome-associated processes, niacin metabolism, and oxidative stress regulation during infection.

In conclusion, we investigated the pathogenic mechanism and potential function of *VdPex30* by constructing knockout mutant strains and using the HIGS strategy. Our results demonstrate that *VdPex30* is significantly upregulated during the early infection stage and that both transient and stable RNAi-mediated silencing of *VdPex30* markedly reduced fungal virulence in host plants, indicating its essential role in disease development. Functional characterization revealed that *VdPex30* is involved in the utilization of multiple carbon sources and regulates responses to cell wall, osmotic, and oxidative stress, highlighting its contribution to fungal adaptability. Protein interaction assays confirmed a specific interaction between VdPex30 and VdMIase, suggesting their cooperative role in modulating niacin metabolism during infection. Taken together, these findings demonstrate that VdPex30 plays roles in the maintenance of carbon metabolism and stress response pathways, thereby affecting fungal growth and virulence. This work expands a novel HIGS target against Verticillium wilt and advances the mechanistic understanding of peroxisome-mediated pathogenesis in *V. dahliae*.

## Data Availability

The transcriptome data generated in this study have been deposited in the NCBI Sequence Read Archive (SRA) under BioProject accession number PRJNA1304425 and have been released and are publicly accessible. All other data supporting the conclusions of this article are included within the article and its supplemental material.

## SUPPLEMENTAL MATERIAL

Supplemental material spectrum.00017-26-s0001.docx Fig. S1 to S5; Tables S1 and S2

ASM does not own the copyrights to Supplemental Material that may be linked to, or accessed through, an article. The authors have granted ASM a non-exclusive, world-wide license to publish the Supplemental Material files. Please contact the corresponding author directly for reuse.
