# Supplementary material for: VdPex30, a peroxisomal membrane protein, is involved in carbon metabolism, stress response, and pathogenicity of Verticillium dahliae
Source: Microbiol Spectr. 2026 May 18;14(7):e00017-26. doi: 10.1128/spectrum.00017-26 (PMC13340144; doi:10.1128/spectrum.00017-26)
Supplement: Supplemental material — Fig. S1 to S5; Tables S1 and S2 [file spectrum.00017-26-s0001.docx]

**VdPex30, a peroxisomal membrane protein, is involved in carbon metabolism, stress response, and pathogenicity of *Verticillium dahliae***

Zhiheng Zhang^1,2^^,3†^, Xiaoqing Liu^1,2,3†^, Wenfang Guo^2,3,4^, Huiming Guo^2,3,4^, Hongmei Cheng^2,3,4^, Xiaoxiao Zhang^1*^, and Xiaofeng Su^2,3,4*^

^1^ Guangxi Key Laboratory of Agro-environment and Agro-product Safety, College of Agriculture, Guangxi University, Nanning 530004, China

^2^ National Key Laboratory of Agricultural Microbiology, Biotechnology Research Institute, Chinese Academy of Agricultural Sciences, Beijing 100081, China

^3^ Key Laboratory of Agricultural Microbiome (MARA), Chinese Academy of Agricultural Sciences, Beijing, 10081, China

^4^ National Nanfan Research Institute, Chinese Academy of Agricultural Sciences, Sanya 572024, China

^†^† Zhiheng Zhang and Xiaoqing Liu contributed equally to this work.

^*^Corresponding author emails: suxiaofeng@caas.cn; zhangxiao0719@126.com.


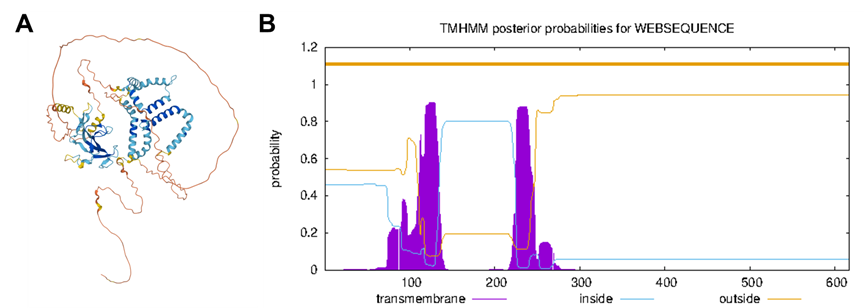


**Fig. S1 Structural analysis of VdPex30. A** The three-dimensional structure of VdPex30 has a core domain rich in α-helices, with few β-sheets and abundant random coils. **B** Prediction of the transmembrane structure of VdPex30. The abscissa represents the position on the sequence, and the ordinate represents the probability of prediction. The horizontal line between 1–1.2 on the ordinate represents the prediction result. The purple line represents the transmembrane region; the blue line represents the inner membrane region; the yellow line represents the outer membrane region.


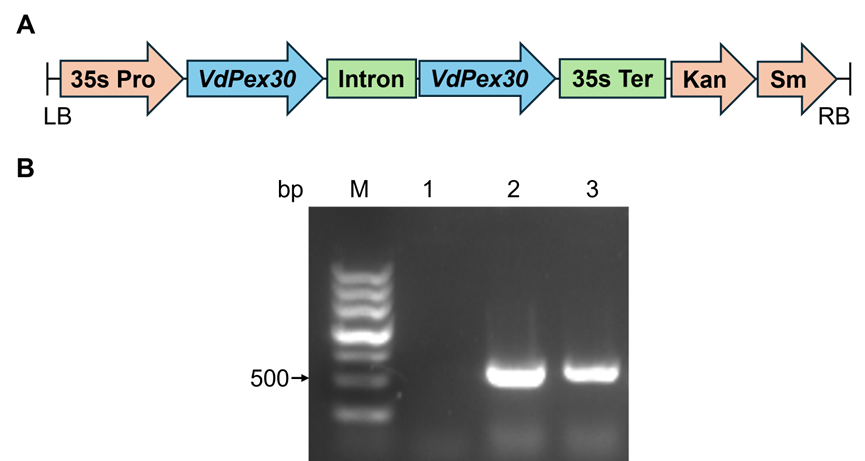


**Fig. S2 Creation and identification of *VdPex30*-RNAi transgenic tobacco. A** Diagram of the *VdPex30*-RNAi vector; **B** Identification of T2 generation *VdPex30*-RNAi positive transgenic tobacco. M: Marker III molecular mass labeling; 1: Amplify *VdPex30* detection fragment in *V. dahliae*; 2-3: Amplify *VdPex30* detection fragment in transgenic tobacco.


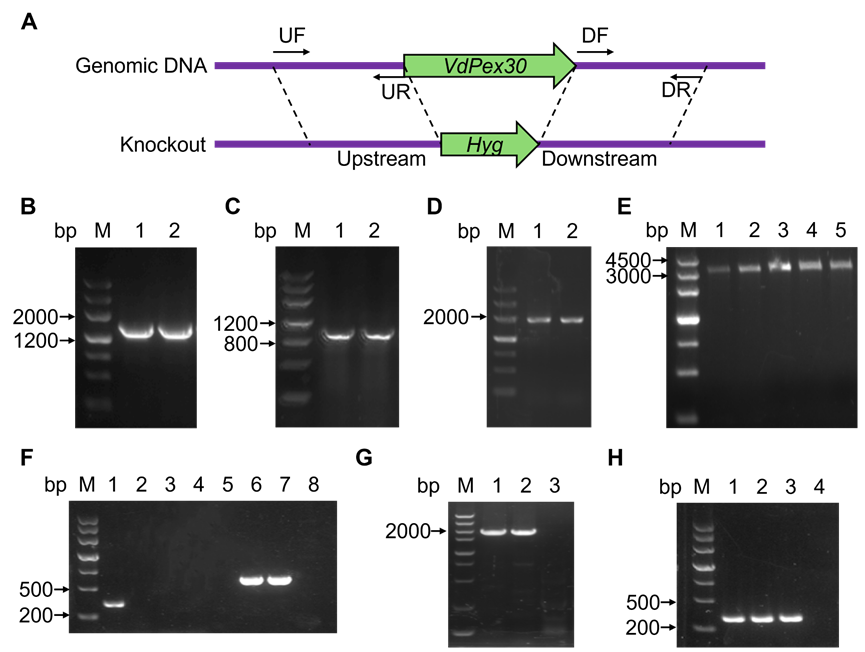


**Fig. S3 Molecular construction and validation of *VdPex30* knockout and complementation strains in *V. dahliae*.** **A** Schematic diagram of the construction of *VdPex30* knockout mutants. **B** Amplification of *VdPex30* upstream fragment. M: Marker III molecular mass labeling; 1-2: PCR identification of *VdPex30* upstream fragment. **C** Amplification of *VdPex30* downstream fragment. M: Marker III molecular mass labeling; 1-2: PCR identification results of *VdPex30* downstream fragment. **D** Amplification of *Hyg* expression cassette. M: Marker III molecular mass labeling; 1-2: PCR identification of *Hyg* expression cassette. **E** Identification of positive single clones. M: Marker III molecular mass labeling; 1-5: Identification of the *VdPex30* homologous recombinant fragment in recombinant plasmid. **F** Identification of Δ*VdPex30* positive transformant with PCR. M: Marker III molecular mass labeling; 1: Amplify *VdPex30* detection fragment in *V. dahliae*; 2-3: Amplify *VdPex30* detection fragment in Δ*VdPex30*; 4: Negative control; 5: Amplify *Hyg* detection fragment in *V. dahliae*; 6-7: Amplify *Hyg* detection fragment in Δ*VdPex30*; 8: Negative control. **G** Identification of *VdPex30* fragment with PCR. M：Marker III molecular mass labeling; 1-2: The amplified fragment of the *VdPex30* full length CDS; 3: Negative control. **H** dentification of Δ*VdPex30-C* positive transformant with PCR. M：Marker III molecular mass labeling; 1: Amplify *VdPex30* detection fragment in *V. dahliae*; 2-3: Amplify *VdPex30* detection fragment in Δ*VdPex30-C*; 4: Negative control.


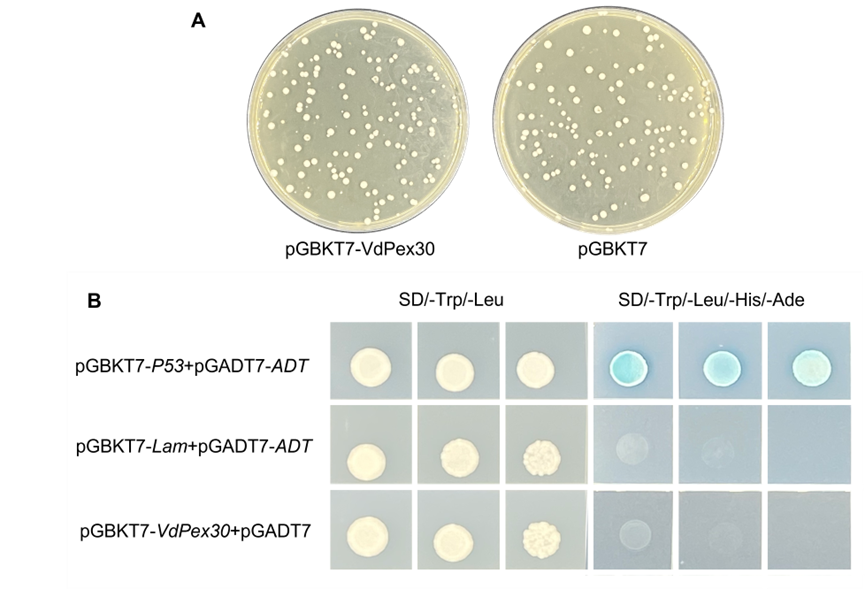


**Fig. S4 Toxicity and Self-Activation Assessments of VdPex30 in Yeast Cells.** **A** Toxicity assay of VdPex30 in yeast cells. The results showed that yeast cells harboring the recombinant plasmid pGBKT7-VdPex30 were able to grow on the SD/-Trp defective medium, with no significant differences in colony size or quantity compared to those containing the empty pGBKT7 vector. **B** Verification of self-activation activity of VdPex30 in the yeast system. Co-transformed yeast cells were cultured on SD/-Trp/-Leu and SD/-Trp/-Leu/-His/-Ade/X-α-Gal media. The yeast cell dilutions in each column, from left to right, are 1/10, 1/100, and 1/1000, respectively.


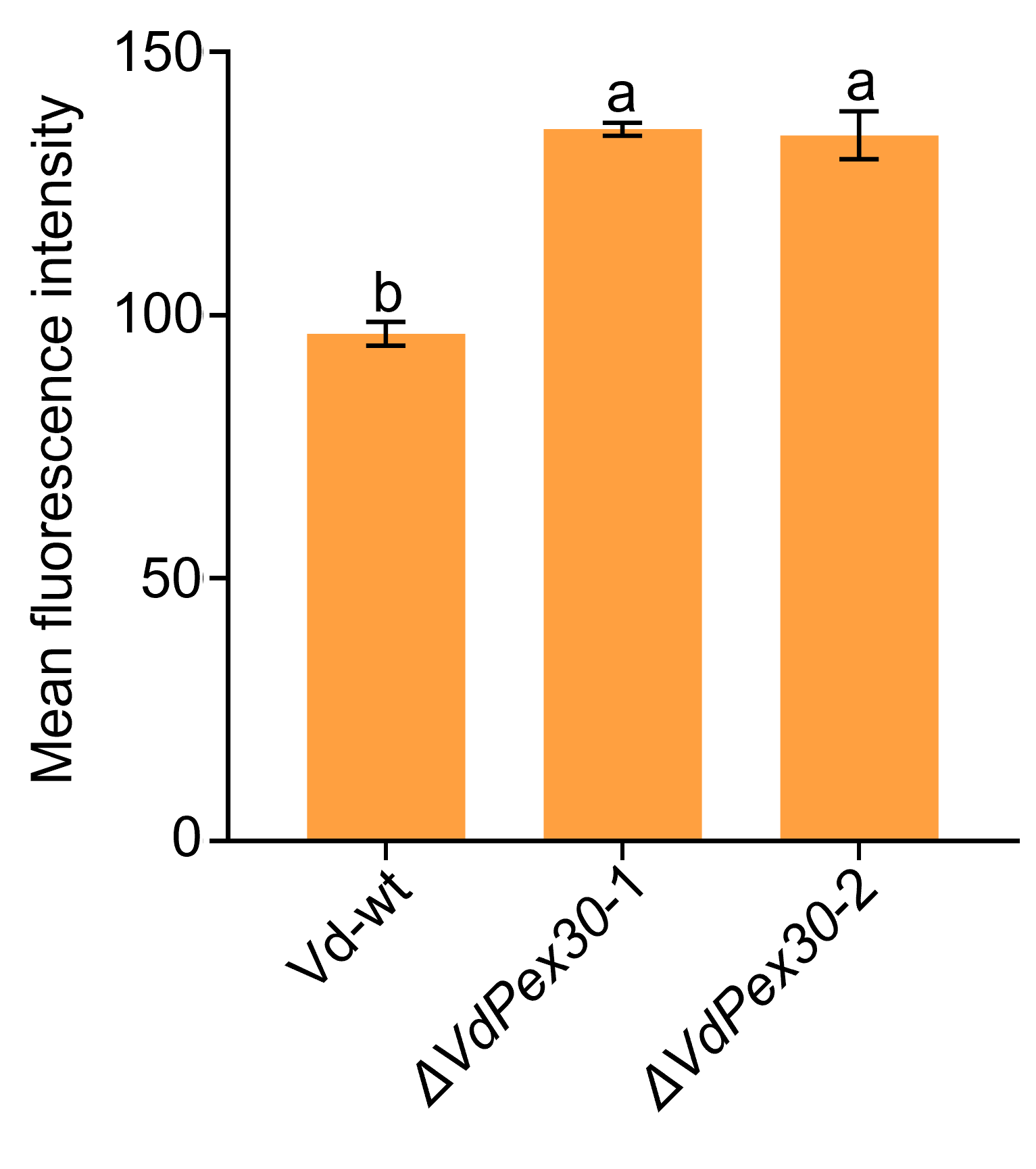


**Figure S5. Quantification of ROS-associated fluorescence intensity.** ROS-associated fluorescence intensity was quantified as mean gray value using ImageJ software. Data represent mean ± SD from three independent biological replicates. Different letters above the bars indicate statistically significant differences among groups as determined by one-way ANOVA followed by Tukey’s multiple comparison test (*P* < 0.05).

**Table S1 Primers used in this study**

| Primer Names | Primer Sequences |
| --- | --- |
| qPCR-Pex30-F | ATTCTCGTACACCAGAAAAGCCCC |
| qPCR-Pex30-R | CGACATCGCCGTAGAGGACGAT |
| Vd-actin-F | GTCCATCTTGCCCTCTTTCCA |
| Vd-actin-R | TCCCTCACCTTCCTTCGGAT |
| VdPex30-HIGS-F | gtgagtaaggttaccgaattcTCGTCATCCGCAGTGCCG |
| VdPex30-HIGS-R | cgtgagctcggtaccggatccCTTGTGGCTCCTCTGACGGA |
| TRV2-test-F | GCGGTTCTTGTGTGTCAAC |
| TRV2-test-R | CAGTCGAGAATGTCAATCTCG |
| Vd-ITS-F | CCGCCGGTCCATCAGTCTCTCTGTTTATAC |
| Vd-ITS-R | CGCCTGCGGGACTCCGATGCGAGCTGTAAC |
| Table S1 (Continued) |  |
| Primer Names | Primer Sequences |
| Ubq-F | AGCTCGGATACGATTGATAACG |
| Ubq-R | GAAGACGAAGAACAAGGGGAAG |
| VdPex30-RNAi-F | aaaaaagcaggctTCGTCATCCGCAGTG |
| VdPex30-RNAi-R | aagaaagctgggtCTTGTGGCTCCTCTG |
| attB-F | GGGGACAAGTTTGTACAAAAAAGCAGGCT |
| attB-R | GGGGACCACTTTGTACAAGAAAGCTGGGT |
| VdPex30-RNAi-test-F | CCCGAACCGACAAGAAACCAGAAGG |
| VdPex30-RNAi-test-R | CCGATGCCGTGACAACATCAGAGT |
| VdPex30-Up-F | GGCGGCAGTCGTGAGGTTTGGT |
| VdPex30-Up-R | gcccaaaaatgctccttcaaTGGTTTCTTGTCGGTTCGGGGT |
| VdPex30-Down-F | ccctgggttcgcaaagataaGTTGCTGAAGAGACGGATTTTG |
| Table S1 (Continued) |  |
| Primer Names | Primer Sequences |
| VdPex30-Down-R | CGTGACTGTGACGGAGGACGAG |
| Hyg-F | TTGAAGGAGCATTTTTGGGC |
| Hyg-R | TTATCTTTGCGAACCCAGGG |
| Nest-VdPex30-F | ggggacaagtttgtacaaaaaagcaggctTGCTGTCTTCCCGTCTTTCTGTCTT |
| Nest-VdPex30-R | ggggaccactttgtacaagaaagctgggtTGTGCTTGGCCTGGTTGTTCTTTAG |
| Hyg-test-F | GAAAAAGCCTGAACTCACCGC |
| Hyg-test-R | TCCGTCAGGACATTGTTGGAG |
| VdPex30-test-F | GCCTACTCGCACTCTTTTCT |
| VdPex30-test-R | TCGACAATCTCGTCCAACGTC |
| VdPex30-pMC-F | GAATTCTGCCACCTTGGATCCATGGCGACCCCAAAACCC |
| VdPex30-pMC-R | GCCCTTGCTCACCATGTCGACCTCGAGACTCATCCTTGCCTCG |
| Table S1 (Continued) |  |
| Primer Names | Primer Sequences |
| VdPex30-BD-F | ATGGCCATGGAGGCCGAATTCATGGCGACCCCAAAACCC |
| VdPex30-BD-R | CCGCTGCAGGTCGACGGATCCTCACTCGAGACTCATCCTTGCC |
| BD-test-F | GGAATTTGTAATACGACTCACTATAG |
| BD-test-R | AAATCATAAATCATAAGAAATTCGCC |
| AD-test-F | GTGAACTTGCGGGGTTTTTCAGTATCTACGATT |
| AD-test-R | ACGAGATCTGGTCGACTAATACGACTCACTATAGGG |
| 01341-AD-F | gccatggaggccagtgaattcATGGGTTCACGTTACAACGACA |
| 01341-AD-R | cagctcgagctcgatggatccCTACTGTTGGCCTTTTAGAAGCG |
| nLUC-VdPex30-F | CGGGGGACGAGCTCGGTACCATGGCGACCCCAAAACCC |
| nLUC-VdPex30-R | TCACTCGAGACTCATCCTTGCC |
| cLUC-VD01341-F | ACGCGTCCCGGGGCGGTACCATGGGTTCACGTTACAACGACA |
| Table S1 (Continued) |  |
| Primer Names | Primer Sequences |
| cLUC-VD01341-R | AGCTCTGCAGGTCGACCTACTGTTGGCCTTTTAGAAGCG |
| YFPN-VdPex30-F | CCGGCGCCGGCGCCGATGGCGACCCCAAAACCC |
| YFPN-VdPex30-R | AACGTTAAGTGAATTTCACTCGAGACTCATCCTTGCC |
| YFPC-VD01341-F | TCCATCACATCACAGATGGGTTCACGTTACAACGACA |
| YFPC-VD01341-R | CGCCGGCGCCGGATCCTACTGTTGGCCTTTTAGAAGCG |
| qPCR-VD06812-F | TTTCAAGAGGTGCTGGTCTC |
| qPCR-VD06812-R | TCTGGACTGCAGTCTCAGG |
| qPCR-VD08724-F | CTCAACTGCCAGTGCATGG |
| qPCR-VD08724-R | TTCCGGGTCTGAACAAGTTG |
| qPCR-VD07524-F | TCAACTGCCAGTGCATGG |
| qPCR-VD07524-R | CTATTTCAATGTGACCAGCTCC |
| Table S1 (Continued) |  |
| Primer Names | Primer Sequences |
| qPCR-VD01315-F | ATCACCACCAGACACATCTTC |
| qPCR-VD01315-R | TGTGTGATTGCTTCTGGGTG |
| qPCR-VD00207-F | AAGAATCATGTCGTCCACCG |
| qPCR-VD00207-R | CGGTGTAGTAGATCTGGATG |
| qPCR-VD06452-F | GGCGACAACTTTTACAAGCAG |
| qPCR-VD06452-R | TTAACGTGGTCGTGCTGGAAA |
| qPCR-VD02087-F | TTGTAGATGACGTTCGCGTG |
| qPCR-VD02087-R | AGTTTGCGTACCATCTGCTAG |
| qPCR-VD00175-F | CGAAGGGGACGATATTGTATTC |
| qPCR-VD00175-R | TGGATGAAAAGGTCACAGGC |
| qPCR-VD09433-F | CACATGTAAGGAGAGTGATCTAGG |
| Table S1 (Continued) |  |
| Primer Names | Primer Sequences |
| qPCR-VD09433-R | CCGAGACCTTGGTACACTC |
| qPCR-VD10248-F | TAGTTCAACTGCCAGTGCATGG |
| qPCR-VD10248-R | CCATGTTGAGAAAGACCATGG |
| qPCR-VD05572-F | CTCCTTGAGTACCGAGACAA |
| qPCR-VD05572-R | TGGACTGCTGCAGCAAGAAT |
| Vd-ITS-F | CCGCCGGTCCATCAGTCTCTCTGTTTATAC |
| Vd-ITS-R | CGCCTGCGGGACTCCGATGCGAGCTGTAAC |

**Table S2 Amplification efficiency of RT-qPCR primer sets**

| Primer set | Slope | R² | Amplification efficiency (%) |
| --- | --- | --- | --- |
| qPCR-Pex30 | -3.15 | 0.992 | 107.9 |
| qPCR-VD07524 | -3.41 | 0.996 | 96.4 |
| qPCR-VD01315 | -3.26 | 0.995 | 102.5 |
| qPCR-VD00207 | -3.37 | 0.993 | 98.1 |
| qPCR-VD06452 | -3.39 | 0.991 | 97.2 |
| qPCR-VD02087 | -3.33 | 0.994 | 99.7 |
| qPCR-VD00175 | -3.44 | 0.989 | 95.2 |
| qPCR-VD09433 | -3.27 | 0.996 | 102.1 |
| qPCR-VD10248 | -3.38 | 0.992 | 97.6 |
| qPCR-VD05572 | -3.42 | 0.991 | 96.1 |
| Table S2 (Continued) |  |  |  |
| Primer set | Slope | R² | Amplification efficiency (%) |
| qPCR-VD06812 | -3.29 | 0.998 | 101.3 |
| qPCR-VD08724 | -3.35 | 0.997 | 98.9 |
